# Spatial Analysis of Expression Patterns Predicts Genetic Interactions at the Mid-Hindbrain Boundary

**DOI:** 10.1371/journal.pcbi.1000569

**Published:** 2009-11-20

**Authors:** Dominik M. Wittmann, Florian Blöchl, Dietrich Trümbach, Wolfgang Wurst, Nilima Prakash, Fabian J. Theis

**Affiliations:** 1 Computational Modeling in Biology, Institute for Bioinformatics and Systems Biology, Helmholtz Zentrum München, German Research Centre for Environmental Health, Munich-Neuherberg, Germany; 2 Zentrum Mathematik, Technische Universität München, Garching, Germany; 3 Molecular Neurogenetics, Institute of Developmental Genetics, Helmholtz Zentrum München, German Research Centre for Environmental Health, Technische Universität München, Deutsches Zentrum für Neurodegenerative Erkrankungen (DZNE), Munich-Neuherberg, Germany; 4 Max Planck Institute of Psychiatry, Munich, Germany; 5 Max-Planck-Institute for Dynamics and Self-Organization, Göttingen, Germany; Memorial Sloan-Kettering Cancer Center

## Abstract

The isthmic organizer mediating differentiation of mid- and hindbrain during vertebrate development is characterized by a well-defined pattern of locally restricted gene expression domains around the *mid-hindbrain boundary* (MHB). This pattern is established and maintained by a regulatory network between several transcription and secreted factors that is not yet understood in full detail. In this contribution we show that a Boolean analysis of the characteristic spatial gene expression patterns at the murine MHB reveals key regulatory interactions in this network. Our analysis employs techniques from computational logic for the minimization of Boolean functions. This approach allows us to predict also the interplay of the various regulatory interactions. In particular, we predict a maintaining, rather than inducing, effect of Fgf8 on *Wnt1* expression, an issue that remained unclear from published data. Using mouse anterior neural plate/tube explant cultures, we provide experimental evidence that Fgf8 in fact only maintains but does not induce ectopic *Wnt1* expression in these explants. In combination with previously validated interactions, this finding allows for the construction of a regulatory network between key transcription and secreted factors at the MHB. Analyses of Boolean, differential equation and reaction-diffusion models of this network confirm that it is indeed able to explain the stable maintenance of the MHB as well as time-courses of expression patterns both under wild-type and various knock-out conditions. In conclusion, we demonstrate that similar to temporal also spatial expression patterns can be used to gain information about the structure of regulatory networks. We show, in particular, that the spatial gene expression patterns around the MHB help us to understand the maintenance of this boundary on a systems level.

## Author Summary

Understanding brain formation during development is a tantalizing challenge. It is also essential for the fight against neurodegenerative diseases. In vertebrates, the central nervous system arises from a structure called the neural plate. This tissue is divided into four regions, which continue to develop into forebrain, midbrain, hindbrain and spinal cord. Interactions between locally expressed genes and signaling molecules are responsible for this patterning. Two key signaling molecules in this process are Fgf8 and Wnt1 proteins. They are secreted from a signaling center located at the boundary between prospective mid- and hindbrain (mid-hindbrain boundary, MHB) and mediate development of these two brain regions. Here, we logically analyze the spatial gene expression patterns at the MHB and predict interactions involved in the differentiation of mid- and hindbrain. In particular, our analysis indicates that *Wnt1* depends on Fgf8 for stable maintenance. A time-course analysis of *Wnt1* expression after implantation of Fgf8-coated beads in mouse neural plate/tube explants experimentally validates our prediction about the interactions between these two key patterning molecules. Subsequently, we demonstrate that available data allows construction of a mathematical model able to explain the maintenance of the signaling center at the MHB. We begin to understand this small aspect of brain formation on a systems level.

## Introduction

During vertebrate development, the central nervous system arises from a precursor tissue called neural plate. Shortly after gastrulation this neural plate is patterned along the anterior-posterior axis into four regions, which continue to develop into forebrain, midbrain, hindbrain and spinal cord. This patterning is determined by a well-defined and locally restricted expression of genes, and by the action of short and long range signaling centers, also called secondary organizers [1]. The development of mid- and hindbrain, for example, is controlled by the activity of the isthmic organizer (IsO) located at the boundary between the prospective mid- and hindbrain, the so-called mid-hindbrain boundary (MHB).

**Figure 1:**
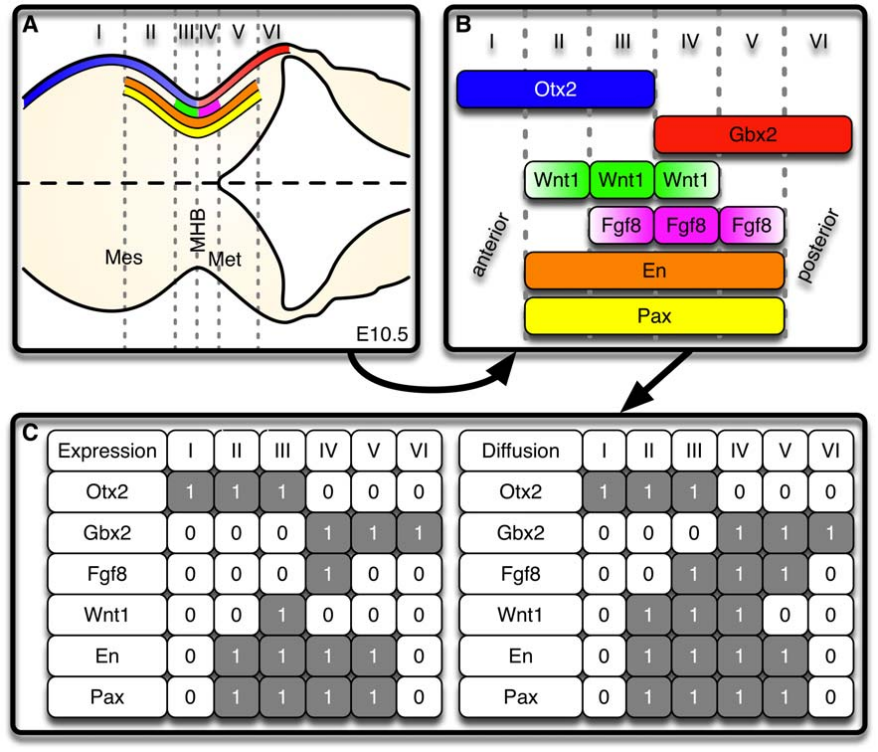
Steady state expression domains of *Otx2* (blue), *Gbx2* (red), *Fgf8* (magenta), *Wnt1* (green), *En* (orange) and *Pax* (yellow) as well as diffusion of Fgf8 and Wnt1 (color gradients) around the MHB at E10.5. (A) Dorsal close-up view of the MHB region in the anterior neural tube of an E10.5 mouse embryo, anterior to the left. The expression pattern of the IsO genes can be subdivided into the six compartments I–VI. (B) Schematic representation of compartments I–VI. The MHB is located between compartments III and IV. (C) Values of the Boolean variables in compartments I–VI. The 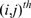 entry indicates if species 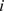 is present (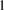) in compartment 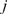 or not (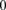). The left-hand matrix (labeled Expression) represents the gene expression pattern. The right-hand matrix (labeled Diffusion) displays protein expression also taking into account diffusion of secreted Fgf8 and Wnt1 protein. Abbreviations: Mes, mesencephalon; MHB, mid-hindbrain boundary; Met, metencephalon.

The IsO is characterized by the localized expression of several transcription and secreted factors. In this contribution, we focus on the following eight IsO genes: *Otx2*, *Gbx2*, *Fgf8*, *Wnt1*, the *Engrailed* genes *En1* and *En2*, which we subsume under the identifier *En*, as well as the *Pax* genes *Pax2* and *Pax5*, which we subsume under the identifier *Pax*. A justification of this selection as well as of the simplifications with respect to *En* and *Pax* is given in the Discussion. Our analysis is based on these gene's steady state expression pattern at E10.5, which is displayed schematically in Figures 1A and 1B. This expression pattern was derived from various *in situ* hybridization experiments, for a review see e.g. [2] and, in particular, Figure 1c therein. All relevant primary research references are also listed in Text S1.

The initial expression of *Otx2* and *Gbx2* in the anterior and posterior neuroectoderm, respectively, defines the position of the prospective fore- and midbrain (*Otx2*+/*Gbx2*−) and hindbrain/spinal cord (*Otx2*−/*Gbx2*+), respectively [3],[4]. The position of the MHB is set at the expression interface of these two transcription factors [5]–[7]. Subsequently, expression of *Wnt1* at the rostral border of the MHB in the caudal midbrain, and of *Fgf8* at the caudal border of the MHB in the rostral hindbrain is initiated independently of a requirement of *Otx2* and *Gbx2* for this process [8],[9]. Fgf8 plays a pivotal role in IsO patterning activity [10],[11] and Wnt1 regulates midbrain development and is required for the maintenance of the MHB [12],[13]. The *Engrailed* genes *En1* and *En2* are both expressed across the boundary [14], yet their expression domains are not fully equivalent. They have been shown to be targets of the *Wnt1* signaling pathway in the midbrain [15] and to regulate the expression of *Fgf8* [16]. Similarly to *Engrailed*, *Pax5* is also expressed across the boundary [17], whereas expression of *Pax2* is restricted to the caudal part of the isthmic organizer [18]. Analysis of *Pax2* -deficient embryos [19] has suggested an essential role of *Pax2* for the induction of *Fgf8*. In short, it has been demonstrated that by E10.5 these genes have become interdependent and form the core module of a regulatory network that guarantees the stable maintenance of their specific expression patterns [2].

Although a great deal of experimental effort has been made, this regulatory network is not yet understood in full detail. For this reason, we now approach this problem on the systems level. In Systems Biology experimentalists and theoreticians make a concerted effort to unravel the functionality of complex biological systems in a holistic fashion. We closely follow the Systems Biology research cycle as proposed in [20] and the manuscript is organized accordingly.

In a first step, we describe a methodology for the inference of regulatory interactions by systematic logical analyses of spatial gene expression patterns. As our information about gene expression at the MHB is of purely qualitative nature we base our analysis on *Boolean models* [21]. These models can, in fact, be seen as the mathematically rigorous representation of qualitative biological knowledge. Their components, henceforth called *species*, can assume only two discrete states, referred to as 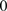 and 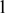. Time is discretized and the state of species 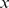 at time 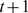 is given by a Boolean update function 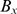, which is a function of the states of the other species at time 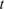. For the determination of these update functions our methodology employs techniques that were originally devised to optimize logic circuits in order to facilitate their hardware implementation.

Applied to the wild-type gene expression pattern at the MHB our method predicts several genetic interactions, which well agree with published data. In this context, an unclarified experimental issue is the reported ectopic induction of *Wnt1* by Fgf8 in gain-of-function (GOF) assays performed *in vitro* and *in vivo* [22]–[25]. As *Fgf8* expression in the anterior neural plate initiates several hours after *Wnt1*, and as *Wnt1* is expressed in a very broad domain at the time most experimental manipulations took place [26],[27], the ectopic maintenance of *Wnt1* expression by Fgf8 might have been mis-interpreted as an ectopic activation. Our analysis indeed predicts that *Fgf8* and *Wnt1* signaling are dependent on each other for stable maintenance, but Fgf8 is not sufficient to ectopically induce *Wnt1* expression. We validate this prediction experimentally by performing a time-course analysis of *Wnt1* expression after Fgf8 -bead implantation into mouse anterior neural plate/tube explants. Thus, our analysis clarifies epistatic relationships at the MHB, especially between the two key patterning molecules Fgf8 and Wnt1.

In a subsequent step, the results of our previous analysis combined with published data allow construction of spatial models that are able to explain the stable maintenance of the characteristic gene expression patterns at the MHB. These patterns are the result of a refinement and sharpening of initially blurred expression domains. Our models are competent to simulate this process. Moreover, we are able to reproduce the phenotypes of various loss-of-function (LOF) experiments even in a correct spatio-temporal order. We conclude with a robustness analysis of our models.

## Results

### Categorization of the expression patterns of key transcription and secreted factors at the MHB

We subdivided the expression patterns of the IsO genes at E10.5 into six compartments I–VI, cf. the dashed lines in Figures 1A and 1B. As is typically the case with Boolean analyses, this preprocessing of biological data was to a certain extent artificial and an over-simplification of biological reality. It was, however, necessary as it allowed us to represent the expression patterns as Boolean matrices (see Figure 1C) which constituted the data basis for our analysis. The morphological entity of the isthmic constriction is located between compartments III and IV. Other than that, this partitioning is based on the expression patterns of the IsO genes and not on morphological properties. Compartments I–III correspond to the prospective midbrain (*Otx2*+/*Gbx2*−) and IV–VI to the prospective hindbrain (*Otx2*−/*Gbx2*+). The boundary compartments III and IV are characterized by the expression of *Wnt1* and *Fgf8*, respectively. We assumed that the secreted Wnt1 and Fgf8 proteins are still present in compartments II and V due to passive or active diffusion [28],[29] (see Discussion), whereas compartments I and VI are devoid of these secreted factors as they are farthest away from the MHB. So, all in all, the six compartments are characterized as follows:

- Only *Otx2* is expressed.
- *Otx2*, *En* and *Pax* are expressed. Secreted Wnt1 protein is present.
- *Otx2*, *En*, *Pax* and *Wnt1* are expressed. Secreted Wnt1 and Fgf8 proteins are present.
- *Gbx2*, *En*, *Pax* and *Fgf8* are expressed. Secreted Wnt1 and Fgf8 proteins are present.
- *Gbx2*, *En*, and *Pax* are expressed. Secreted Fgf8 protein is present.
- Only *Gbx2* is expressed.

The crucial point is that this expression pattern is stably maintained by a regulatory network. In the next section we demonstrate that key genetic interactions of this network can be obtained already by analyzing only this spatial information.

Information about the expression patterns of the IsO genes can also be found in our *IDGenes* database (http://www.helmholtz-muenchen.de/idgenes). Here we compiled detailed information about gene expression domains at the MHB in anterior–posterior, dorso–ventral and medio–lateral directions. This information is complemented by already published interactions between the IsO genes. Using a web interface it is possible to visualize regional distinctions in the expression of genes within the embryonic mouse neural tube in a three dimensional manner (see Text S1).

### Logical analysis of gene expression patterns

So far, information about genetic interactions at the MHB has been obtained mainly from the analyses of gene expression patterns (by *in situ* hybridization) and epistatic relationships between these genes in GOF and LOF mutant mice. The task of elucidating gene regulation at the MHB therefore leads to the theoretical challenge of inferring as much information as possible about the structure of multi-cellular regulatory networks from their spatial expression patterns. To this end, we now describe a methodology for the inference of regulatory interactions by systematic logical analyses of spatial gene expression patterns.

**Figure 2:**
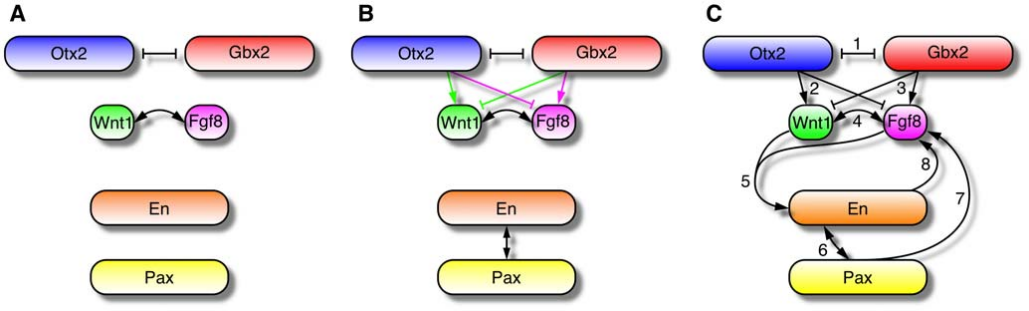
Regulatory networks between the IsO genes. (A) Necessary interactions. Any valid network includes a mutual inhibition of *Otx2* and *Gbx2* as well as a mutual activation of *Fgf8* and *Wnt1*. (B) Four minimal networks maintain the steady state expression pattern of the IsO genes. They are obtained by including either of the two green and either of the two magenta interactions. (C) Regulatory network obtained from literature data. It includes the following interactions: *(1)* The mutual repression of *Otx2* and *Gbx2* [5],[6]. *(2)* The positive regulation of *Wnt1* and the negative regulation of *Fgf8* by *Otx2* [3],[5],[93]. *(3)* The positive regulation of *Fgf8* and the negative regulation of *Wnt1* by *Gbx2* [4],[6],[25]. *(4)* The maintenance of *Fgf8* by Wnt1 [10] and, vice versa, the maintenance of *Wnt1* by Fgf8 as demonstrated herein and by [11]. *(5)* The synergy between Fgf8 and Wnt1 in the maintenance of *En* [15],[23],[26],[27]. *(6)* The mutual activation of *En* and *Pax* [25],[31],[32]. *(7)* The induction of *Fgf8* by *Pax* [19],[94]. *(8)* The positive regulation of *Fgf8* by *En* [16].

These analyses are based on a Boolean model whose species denote, in our case, the (groups of) genes *Otx2*, *Gbx2*, *Fgf8*, *Wnt1*, *En* and *Pax*. Figure 1C shows a Boolean representation of their expression patterns around the MHB. We already remarked that the stable maintenance of these patterns is a key function of the regulatory network between the IsO genes. Taking this into account, we performed logical analyses of the matrices from Figure 1C in order to determine key interactions within this network (see Methods). Our first approach was to determine all necessary interactions. Here the idea is that each border in the spatial expression pattern of a gene is caused by a change of at least one of the gene's regulators at this position. Now, if only one other gene changes its expression at this position, we can be sure that there is an indispensable interaction between these two genes. This analysis shows that the maintenance of the MHB requires a mutual inhibition of *Otx2* and *Gbx2* as well as a mutual activation of *Fgf8* and *Wnt1* (see Figure 2A).

In a second approach, we determined as simple a Boolean update function as possible for each species such that the expression pattern is still maintained. This analysis is based on the assumption that the thus obtained minimal network is the core module of the real network. However, we cannot be sure that all the interactions are indeed necessary. The following minimal functions could be derived using a technique called Karnaugh-Veitch maps (see Methods and, for further details on Karnaugh-Veitch maps, Text S1):
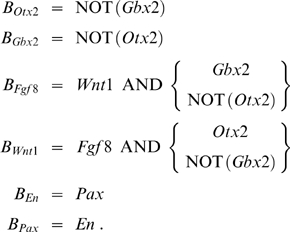
(1)


In the case of *Fgf8* and *Wnt1* two equally minimal expressions could be found, indicated by the bracketed factors, of which at least one has to be included. The corresponding networks are shown in Figure 2B. We point out that equations (1) were derived solely from the wild-type expression patterns shown in Figure 1. Below, we will extend the minimal networks using additional literature knowledge (see Figure 2C).

The minimal networks from Figure 2B show that, in addition to the necessary mutual inhibition of *Otx2* and *Gbx2*, these two transcription factors have antagonistic effects on *Fgf8* and *Wnt1* expression. This agrees well with experimental results showing that opposing interactions between *Otx2* and *Gbx2* are required for the positioning and refinement of the MHB [8],[30]. Furthermore, a mutual positive regulation between *En* and *Pax* was predicted which is well supported by experimental results from [25],[31],[32]. Finally, with respect to the necessary mutual activation of *Fgf8* and *Wnt1*, this analysis revealed that *Fgf8* and *Wnt1* require each other for their stable maintenance but are not sufficient to induce each other's expression, as the respective update rules contain at least one additional factor. The early loss of *Fgf8* expression in 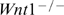 mutants by nine-somites [10] indeed suggests that Wnt1 is directly required for the maintenance of *Fgf8* expression [33]. We subsequently set out to validate also the prediction that Fgf8 is, in turn, necessary for the maintenance but not sufficient for the induction of *Wnt1* expression.

### Fgf8 maintains but does not induce *Wnt1*

**Figure 3:**
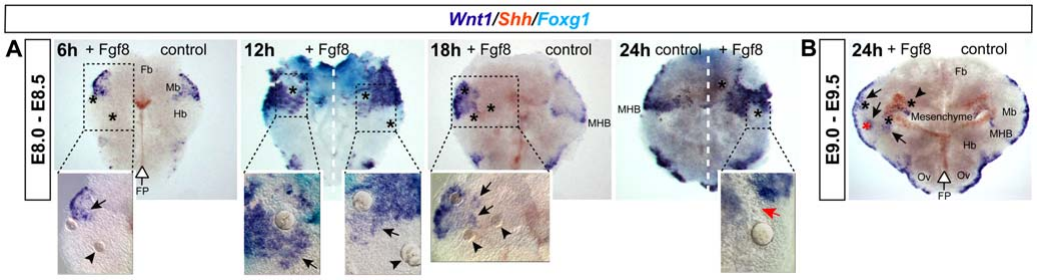
*Wnt1* expression is maintained in the midbrain and at the MHB by Fgf8 -coated beads. (A) E8.0–E8.5 (4–6 somites) mouse anterior neural plate explants (cf. upper Figure 5A) were incubated for 6, 12, 18 and 24 hours (h) after implantation of Fgf8 -coated beads (asterisks) and prior to fixation and detection of *Wnt1* (dark blue), *Shh* (red) and *Foxg1* (*Bf1*, a forebrain marker; light blue) expression by whole mount *in situ* hybridization (WISH). High magnifications of the boxed areas in the overviews are shown below. Repression of endogenous *Wnt1* (red arrow) around an Fgf8 -bead located in the presumptive rostral hindbrain close to the MHB is apparent in the 24 h explant. (B) E9.0–E9.5 (20–25 somites) mouse anterior neural tube explant incubated for 24 h after implantation of Fgf8 -coated beads (asterisks) and prior to fixation and detection of *Wnt1* (dark blue) and *Shh* (red) expression. The ectopic *Wnt1* expression around the Fgf8 -coated bead marked by a red asterisk might have been maintained during the 24 h incubation period. *Shh* expression in the FP (open arrow) or a white broken line indicate the position of the ventral midline of the neural plate/tube in the explants. Beads were implanted unilaterally except for the 12 h explant. The contralateral side served as control. The control side shows the *Wnt1* expression pattern under normal (unmanipulated) conditions, the Fgf8 -treated side shows the changes in *Wnt1* expression after bead implantation. Ectopic *Wnt1* expression around Fgf8 -coated beads is indicated by black arrows. Beads that did not induce ectopic *Wnt1* expression are indicated by black arrowheads. Abbreviations: Fb, forebrain; FP, floor plate; Hb, hindbrain; Mb, midbrain; MHB, mid-hindbrain boundary; Ov, otic vesicle.

To find out if Fgf8 only maintains or de novo induces *Wnt1* expression *in vivo*, we performed a time-course analysis of *Wnt1* expression after implantation of Fgf8 -coated beads into mouse anterior neural plate (E8.0–E8.5) or tube (E9.0–E9.5) explants (see Figure 3). In E8.0–E8.5 anterior neural plate explants (Figure 3A), *Wnt1* expression retracted from its originally broad expression domain in the prospective midbrain (control side of explant incubated for 6 h) and was confined to the dorsal midline of the midbrain and to the rostral border of the MHB in the control side of explants incubated for 18 and 24 h. Fgf8 -coated beads implanted within or close to the endogenous *Wnt1* expression domain in the midbrain (*Otx2*+/*Gbx2*− territory, not shown) maintained *Wnt1* expression within but not outside of this domain over 24 h (compare with contralateral control side of the explants). Notably, Fgf8 -coated beads placed outside the endogenous *Wnt1* expression domain in the rostral hindbrain (*Otx2*−/*Gbx2*+ territory, not shown) or forebrain (*Otx2*+/*Gbx2*− territory) did not induce ectopic *Wnt1* transcription at any time-point analyzed, as predicted by our logical analysis. When these experiments were performed with E9.0–E9.5 anterior neural tube explants incubated for 24 h (as in [23],[25]) (Figure 3B), ectopic expression of *Wnt1* was only observed around Fgf8 -coated beads that were implanted 24 h before close to the endogenous *Wnt1* domain in the midbrain. This result shows that the ectopic *Wnt1* expression observed by [23],[25] is due to the maintenance but not de novo induction of *Wnt1* transcription by Fgf8 . Therefore, it is in agreement with our predicted update rule (1) for *Wnt1*, which implies that *Wnt1* expression requires Fgf8 for stable maintenance, but that Fgf8 is not sufficient to induce *Wnt1* ectopically.

### Setup of a regulatory network and model of the MH

The previous prediction of genetic interactions was based solely on spatial gene expression data from the MHB at E10.5. Here, we complemented this by a careful literature mining for further experimentally validated interactions. Thus, we extended the minimal networks from Figure 2B to a literature-based regulatory network between *Otx2*, *Gbx2*, *Fgf8*, *Wnt1*, *En* and *Pax*. Its topology is shown in Figure 2C; references for all included interactions are given in the caption and can also be retrieved from the *IDGenes* database.

The function of this gene regulatory network is threefold. First, it has to maintain the characteristic expression pattern of the MHB. Second, it has to ensure that once expression of a gene has been induced its expression domain is correctly positioned. Third, it has to account for the experimentally observed sharpening of the expression domains and of the whole boundary. In order to check whether these functionalities are ensured, we constructed dynamic models of the interaction network. These are described in the remainder of this section; the *in silico* experiments that we carried out for the analysis are then explained in the next section. Mathematical details about the modeling process can be found in the Methods; implementations of the models are provided in Dataset S1 and Dataset S2.

In a first step, we derived Boolean update rules from the regulatory network. The resulting Boolean model was then implemented in six linearly ordered compartments that correspond to the subdivision of the expression patterns shown in Figure 1. The compartments are able to communicate via *Fgf8* and *Wnt1* signaling. Thus we obtained a multi-compartment Boolean model of the MHB. Using this model it was easy to check if the expression patterns from Figure 1 are maintained as a steady state.

To analyze if the regulatory network is also able to correctly position the expression patterns of the IsO genes after their induction, the Boolean model was transformed into a multi-compartment ordinary differential equation (ODE) model. The variables are now no longer coarse-grained into discrete states but assume continuous values. Continuous variables are necessary as we cannot assume that a gene is induced already to its full expression level. Rather the model should amplify a gene's expression at the right positions while downregulating it elsewhere.

The ODE model is still based on the six compartments shown in Figure 1. In order to test for the third functionality, however, a continuously resolved spatial coordinate was necessary. To this end, the multi-compartment model was transformed into a system of partial differential equations (PDE). These equations describe the change of the species in space and time taking into account production, decay and, in the case of Fgf8 and Wnt1 , spreading of secreted protein. For the latter a diffusion-based mechanism is assumed (see Discussion). The spatial dimension of the PDE model is no longer coarse-grained into compartments but continuously resolved. This allowed for the analysis of continuous spatial processes, such as the contraction and expansion of expression domains or the sharpening of boundaries.

### *In silico* model verification

We analyzed if the network constructed in the previous section indeed fulfills its three functions. To this end, we performed several *in silico* experiments on the three models that we derived from the network.

#### Maintenance of steady state expression patterns

A simple simulation of the multi-compartment Boolean model (not shown) confirmed that the regulatory network stably maintains the expression patterns from Figure 1. The update policy (synchronous or asynchronous) is irrelevant as it does not influence the model's steady state behavior.

#### Correct positioning of expression domains

**Figure 4:**
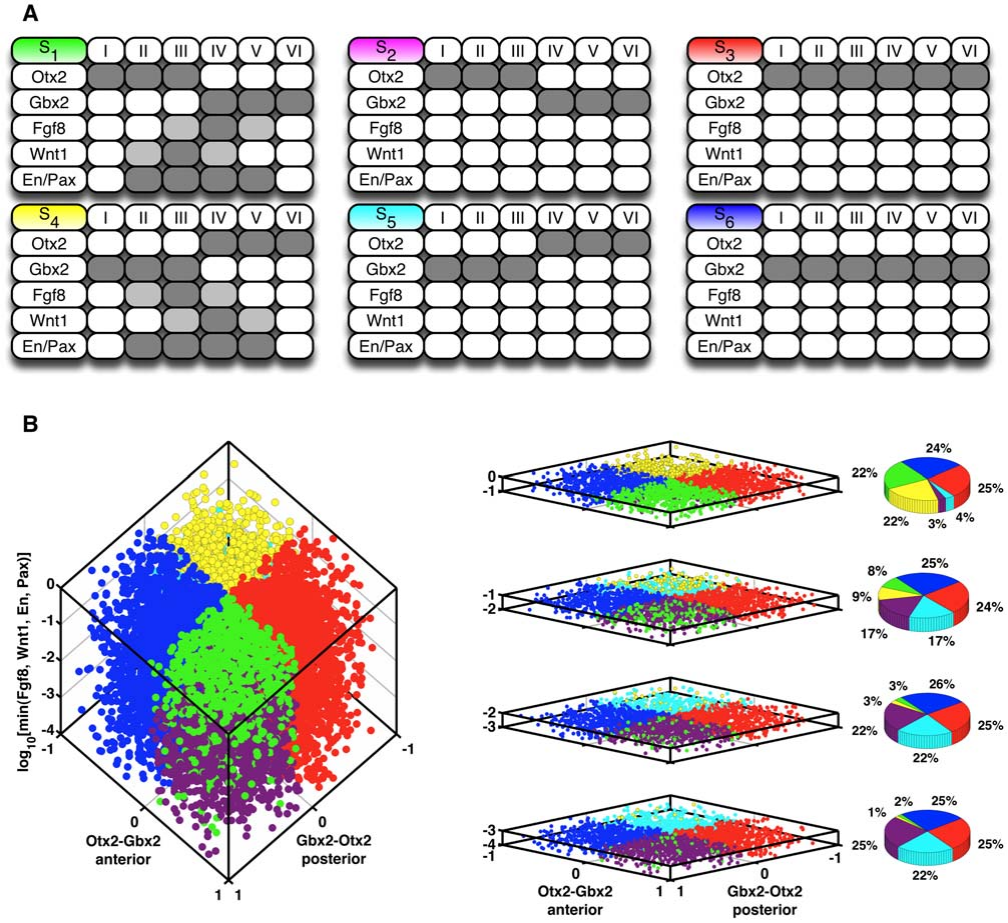
Simulations of the continuous multi-compartment model. In lack of quantitative data ad-hoc parameters were used. For different initial conditions 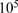 numerical simulations of the model were run until convergence. Each initial condition was determined by eight values — the levels of *Otx2* and *Gbx2* on the anterior as well as posterior side of the boundary and the levels of *Fgf8*, *Wnt1*, *En* and *Pax*, whose initial expression domains were all set to the four central compartments. Hence, the initial conditions were not biased towards the desired expression pattern of these genes. (A) Classes of steady states reached by the model. Dark grey: expression, light grey: no expression but diffusion of secreted protein, white: neither expression nor diffusion. (B) On the left-hand side a scatter plot of the reached steady states is shown. The X- and Y-axes mark the difference between the initial concentrations of *Otx2* and *Gbx2* in the anterior and posterior side of the neural plate/tube. The Z-axis marks the minimum of the initial concentrations of *Fgf8*, *Wnt1*, *En* and *Pax* on a log-scale. Each marker in the scatter plot corresponds to one of the 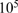 model simulations. Its spatial position is determined by the initial conditions of the model variables, its color by the steady state that was reached, cf. A. For better visualization the cube on the left-hand side was sliced up horizontally (middle column). The distribution of the six steady states from A in each slice is shown on the right-hand side. Details about the ad-hoc parameters, the sampling of the initial conditions and the classification of the steady states can be found in Text S1.

For different initial conditions, 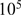 numerical simulations of the multi-compartment ODE model were run until convergence and then classified accordingly to the steady state they reached (see Figure 4 as well as Text S1 for technical details). Each initial condition was determined by eight values — the levels of *Otx2* and *Gbx2* on the anterior as well as posterior side of the boundary and the levels of *Fgf8*, *Wnt1*, *En* and *Pax*, whose initial expression domains were all set to the four central compartments. This guarantees that the initial conditions were not biased towards the desired expression pattern of these genes. In all simulations a steady state was reached that can be assigned to one of the six classes 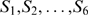 shown in Figure 4A. The states 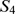 and 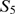 are the reflections of 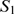 and 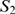, respectively, across the MHB. Up to this symmetry, 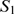 and 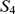 correspond to the wild-type gene expression pattern at the MHB (see Figure 1). In 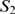 and 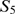 an interface between *Otx2* and *Gbx2* is still established, expression of the other IsO genes, however, is lost. Finally, in 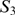 and 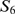 no boundary at all exists, but either *Otx2* or *Gbx2* is expressed along the whole neural tube. The distribution of the six steady states over the 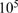 runs is shown in Figure 4B.

In our network *Otx2* and *Gbx2* are upstream of all other species and mutually inhibiting each other, cf. Figure 2C. For this reason, we expected the steady states of *Otx2* and *Gbx2* to be solely dependent on the initial conditions of these two species in a compartment. More precisely, we expected that in each compartment the initially dominating species will continue to be expressed and ultimately repress the other. In our simulations this expectation was met. First of all, we observed that in none of the steady states these two genes are coexpressed in any compartment. Moreover, from the pie charts in Figure 4B one can see that 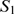 together with 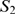 (*Otx2* – *Gbx2* interface), 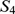 together with 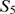 (*Gbx2* – *Otx2* interface), 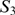 (only *Otx2*) and 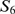 (only *Gbx2*) were each reached in about 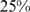 of the simulations. In the cases in which a boundary was created, the distribution within the groups 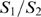 and 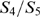 crucially depended on the initial concentrations of the other IsO genes. Again from Figure 4B we see that the share of 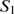 and 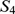, i.e. of the states where the other IsO genes are expressed in a correct spatial pattern, increased with the minimum of their initial expression levels. This agrees well with the experimental observation that these genes are dependent on each other for stable maintenance, and that, when one of them is missing, the expression of the remaining genes is extinguished over time [2],[19]. In other words, a missing expression cannot be compensated for by high expression levels of the other genes.

This result clearly shows the potential as well as the limitations of our model. As soon as there was a predominance of *Otx2* expression in the mid- and of *Gbx2* expression in the hindbrain as well as sufficient (unpatterned) expression of *Fgf8*, *Wnt1*, *En* and *Pax* around the boundary, the model was able to establish and maintain the correct pattern of expression domains (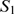). It was, however, unable to induce expression of *Fgf8*, *Wnt1*, *En* and *Pax* (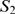). Being symmetric with respect to the boundary, it was naturally also not able to determine the correct orientation of the compartments, but depended on the bias set by the initial conditions (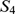 and 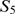). If this bias was missing no boundary was created at all (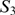 and 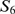).

#### Refinement of expression patterns

**Figure 5:**
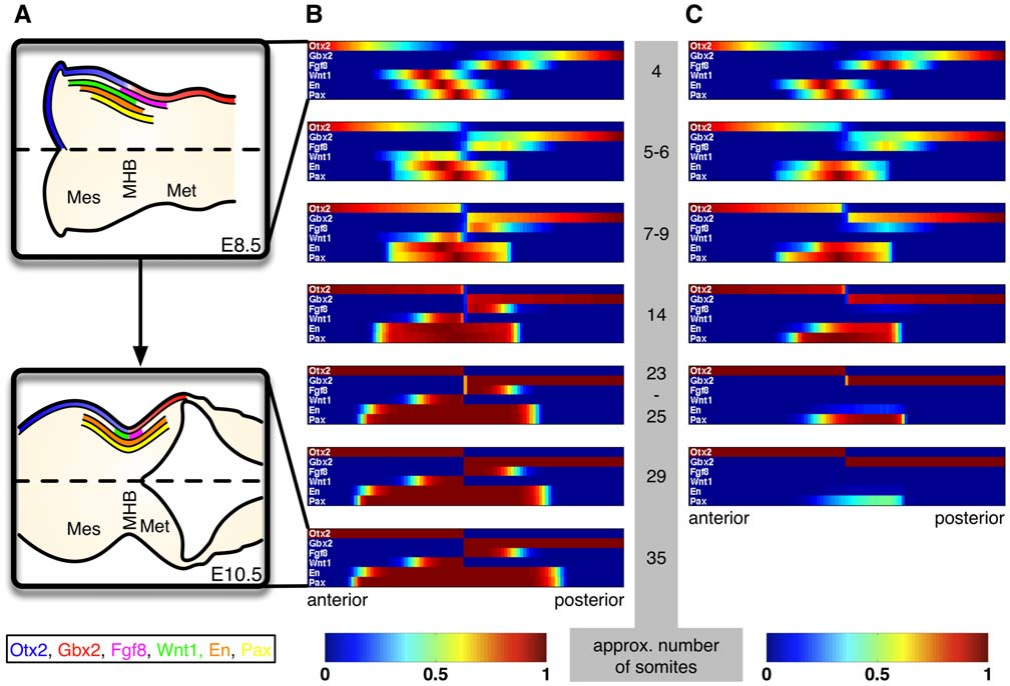
Time-courses of the IsO genes under wild-type and knock-out conditions. (A) Expression pattern of the IsO genes at E8.5 (upper figure) and at E10.5 (lower figure). The initially blurred expression domains are refined and a sharp boundary is established. (B, C) Simulations of the PDE model. Initial conditions were chosen to resemble the expression pattern at E8.5, cf. upper Figure A. In lack of quantitative data ad-hoc parameters were used (see Text S1). (B) Simulated gene expression domains under wild-type conditions. The refinement and sharpening of the expression domains is clearly visible. Finally an expression pattern similar to lower Figure A is established. (C) Simulation of a 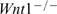 mutant. The expression of *Fgf8*, *En* and *Pax* is lost over time. In particular, the expression of *Fgf8* and *En* agrees well with the time-courses described in [10],[26], cf. section Simulation of LOF experiments. From this comparison we obtain a time-scale of approximately 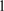 somite per every 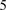 time units. Accordingly, in B and C model simulations are shown at time-points 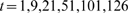 and (only in B) 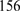. Abbreviations: Mes, mesencephalon; MHB, mid-hindbrain boundary; Met, metencephalon.

The blurred expression domains of the IsO genes at E8.5 as well as their sharp expression domains and the clearly demarcated boundary at E10.5 are shown in upper and lower Figure 5A, respectively. Simulations of the PDE model showed that it is able to reproduce this development (see Figure 5B). Starting from initial conditions chosen to resemble the expression domains at E8.5 the model established the characteristic expression pattern at E10.5 (see Text S1 for technical details and Video S1 for a movie animation). This demonstrates that our model — although not accounting for its initial induction — also explains the refinement and sharpening of gene expression patterns at the MHB. In lack of quantitative data, ad-hoc parameters were used for the simulation. Consequently, the time-scale in our model is, at first, undefined. Below, we use experimentally observed time-courses from a LOF experiment to map the model's time-scale onto the somitogenesis clock.

### Simulation of LOF experiments

A key feature of the IsO genes studied here is their tight and indispensable interaction for the maintenance of their own expression and of the MHB during midgestational stages, as revealed by the analysis of LOF mouse mutants for these genes [2],[19]. Simulations showed that our model is able to reproduce these experiments. We discuss 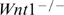 mutants as a showcase. From [10],[26] the following time-course of *Fgf8* and *En* expression in these mutants can be reconstructed:

**4 somites:** *En* and *Fgf8* expression indistinguishable from wild-type

**5–6 somites:** Reduction of the *En* domain in the midbrain by loss of anteriormost *En* expression, *Fgf8* expression indistinguishable from wild-type

**7–9 somites:** *En* expression restricted to hindbrain, *Fgf8* expression markedly reduced

**14 somites:** *En* still expressed in hindbrain, *Fgf8* expression completely abolished

**23–25 somites:** *En* expression reduced to a small domain in the hindbrain

**29 somites:** *En* expression completely abolished

An *in silico* simulation demonstrated that our PDE model is able to reproduce this phenotype even in a correct spatio-temporal order (see Figure 5C). Except for *Wnt1*, initial conditions were chosen to resemble the expression domains of the IsO genes in the wild-type at E8.5 (see upper Figure 5A). A comparison of the model simulation with the above time-course indicated a linear relationship between the model's time-scale and the somitogenesis clock, where approximately 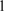 somite is formed per every 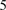 time units.

Similar simulations of 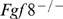, 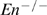 and 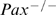 mutants can be found in Text S1.; movie animations of all *in silico* LOF experiments are provided in Video S2, Video S3, Video S4 and Video S5. These computational experiments further corroborate our model and give additional evidence that it indeed captures the gene regulatory network at the MHB to a large extent.

### Parameter and robustness analysis

So far, we used ad-hoc parameter estimates for the simulation of the ODE and PDE model. We now analyse how robust the obtained results are under perturbations of the kinetic parameters.

**Figure 6:**
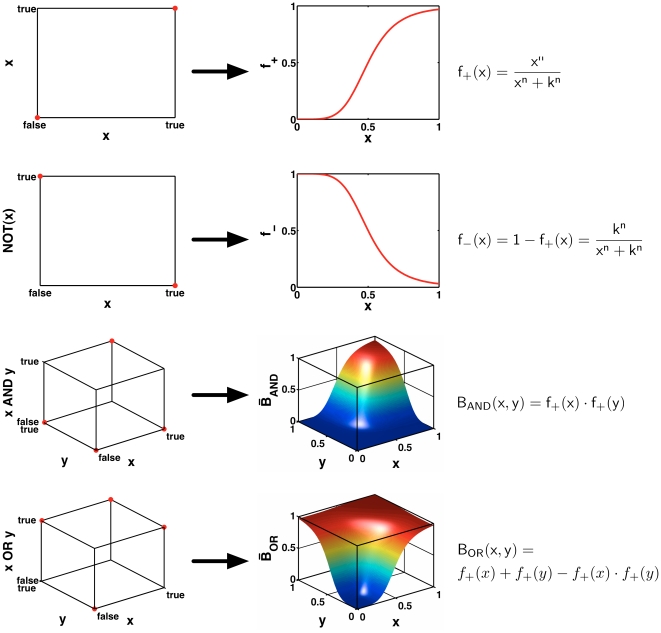
Transformation of Boolean update rules into continuous activation functions. Boolean step functions are replaced by positive and negative sigmoid Hill functions. The continuous homologues of the **AND** and **OR** gates are obtained by interpolating the Boolean functions (cf. the left-hand and middle column) resulting in the mathematical expressions shown in the right-hand column. For mathematical details of this transformation see [92].

In the ODE model, each interaction is modeled by a sigmoid Hill kinetic and the effects of different regulators on one species are combined accordingly to the Boolean logic (see Methods and Figure 6). Hill functions are well suited to model transcriptional gene regulation. They contain two parameters with clear-cut biological meanings: One measuring the cooperativity of the transcription factor-promotor binding, and one measuring the affinity of the promotor for the transcription factor. Additionally, we have one parameter per species describing its life-time.

The network from Figure 2C can be divided into an upstream part consisting of the mutual inhibition between *Otx2* and *Gbx2* and a downstream part consisting of the positive maintenance loop between *Fgf8*, *Wnt1*, *En* and *Pax*. The mutual inhibition of two genes, sometimes referred to as a toggle or switch, is a frequently found motif in regulation of cell specification and has been studied *in vivo* as well as *in silico* [34]. In principle, two stable steady states are possible: The two states where one species is fully expressed and repressing the other, which is consequently not expressed. The corresponding basins of attraction depend on the parameters governing the two inhibitions. In the case of symmetrical parameters, the initially dominating species wins and we have two equally large basins of attraction. Differing parameters destroy this symmetry and the switch becomes unbalanced. For sufficiently large differences the switch exhibits an essentially monostable behavior: The more strongly inhibited species will ultimately be repressed irrespective of the initial conditions (see also Text S1). In our model the switch between *Otx2* and *Gbx2* is responsible for the division of the neural tube into an *Otx2*+/*Gbx2*− and an *Otx2*−/*Gbx2*+ territory. Hence a robust patterning of the neural tube requires the parameters governing the *Otx2* – *Gbx2* switch to be of the same magnitude.

**Figure 7:**
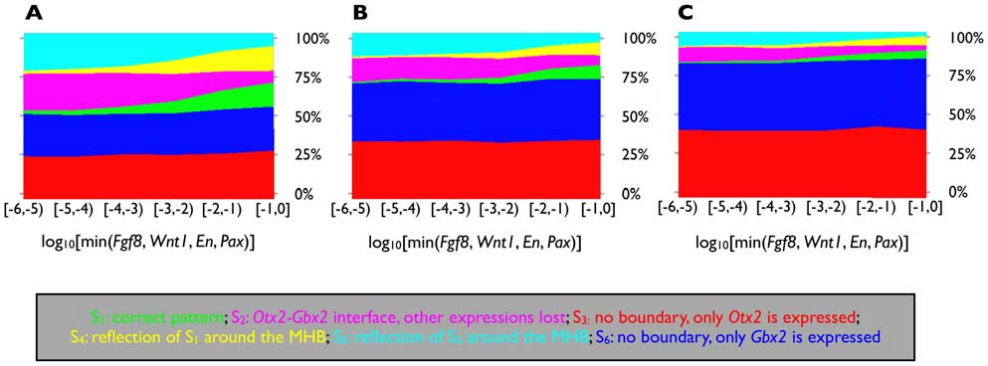
Parameter robustness analysis. The computational experiment from Figure 4 was repeated. However, this time for each run a different parameter set was used. Initial conditions were sampled essentially as described in Figure 4; to account for the wide range of parameter values, the range of the initial conditions of *Fgf8*, *Wnt1*, *En* and *Pax* was enlarged to 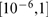. For the parameters governing the *Otx2* – *Gbx2* switch three different sampling schemes were used. (A) The parameters were set to their ad-hoc estimates already used in Figure 4. (B) They were sampled randomly from a 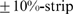 around the ad-hoc values. (C) They were sampled randomly as the other parameters. The continuous multi-compartment model was simulated until convergence and the reached steady state was classified accordingly to Figure 4A. The runs were binned accordingly to the minimum of the initial expression levels of *Fgf8*, *Wnt1*, *En* and *Pax*. The bins are shown on the X-axes. For each bin the distribution of the six steady states from Figure 4A is shown on the Y-axes. Details about the sampling of the parameters and initial conditions as well as about the classification of steady states are provided in Text S1.

We repeated the computational experiment from Figure 4, this time using a different set of parameters for each run. The parameters in the downstream part were sampled randomly over a wide range. For the parameters describing the crucial, boundary forming *Otx2* – *Gbx2* switch, three different sampling schemes were used, cf. Figures 7A–7C. In subfigure A the parameters were set to their ad-hoc estimates already used in Figure 4. In subfigure B they were sampled randomly from a 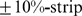 around the ad-hoc values. In subfigure C, they were sampled randomly as the other parameters. Technical details can be found in Text S1. We observed that the results from Figure 4 are reproduced robustly under parameter changes. For all three sampling schemes the shares of 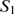 and 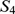, i.e. of the states where all IsO genes are, up to symmetry, expressed in a correct spatial pattern, increased with the minimum of the initial expression levels of *Fgf8*, *Wnt1*, *En* and *Pax*, cf. the green and yellow areas in Figures 7A–7C. Concomitantly, the shares of 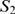 and 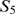, i.e. of the states where an interface between *Otx2* and *Gbx2* is established but expression of the other IsO genes is lost, decreased, cf. the magenta and cyan areas. The shares of 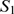 together with 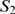 (*Otx2* – *Gbx2* interface, green and magenta areas) and of 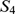 together with 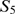 (*Gbx2* – *Otx2* interface, yellow and cyan areas) were independent of the gene's initial expression levels. The difference between the sampling schemes is that with increasing randomness in the *Otx2* – *Gbx2* switch parameters the shares of 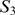 (no boundary, only *Otx2* is expressed) and 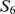 (no boundary, only *Gbx2* is expressed) got larger, cf. the red and blue areas. This was due to the increasingly biased behavior of the switch. Still, for 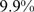 of the randomly sampled parameters in Figure 7C, a — up to symmetry — correct expression pattern was established. As we have a total of 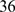 parameters, this implies that on average a random choice of parameter value has a 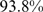 chance of being compatible with the desired behavior (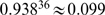).

In addition to the parameters from the ODE model, the PDE model also contains parameters describing the production and decay as well as the diffusion of the secreted Fgf8 and Wnt1 proteins. The diffusion constants essentially determine the length of the *Fgf8*, *Wnt1*, *En* and *Pax* expression domains and thus affect the model's steady state. However, they can be varied over a wide range without changing the qualitative behavior of the model (see Text S1). With respect to the production and decay rates we note that the decay rates need to be sufficiently small compared to the production rates in order to allow for diffusion. The ad-hoc values of these parameters were chosen such that deviations of 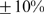 are tolerated. Higher decay and lower production rates lead to a complete loss of *Fgf8*, *Wnt1*, *En* and *Pax* expression over time.

Using ad-hoc estimates for the parameters, we also investigated the effect of changes in the initial conditions from Figure 5B on the PDE model (see Text S1). As expected, we observed that the initial conditions have a crucial effect on the position of the MHB along the anterior-posterior axis of the neural tube. However, up to this shift, the model's steady state expression pattern was robustly preserved under perturbations of the initial conditions. In particular, the expression domains of *En* and *Pax* became centered around the MHB irrespective of possible asymmetries in their initial conditions.

## Discussion

### Selection of IsO genes and simplification of expression domains

Our main objective in this contribution is to elucidate epistatic relationships at the MHB on the transcriptional level. Initially and since ours is the first approach to modeling this biological system, we preferred to include only the eight best-studied and most important IsO genes that are known to constitute the core of the regulatory network maintaining the MHB: the transcription factors *Otx2*, *Gbx2*, *En1*, *En2*, *Pax2* and *Pax5* as well as the secreted factors *Fgf8* and *Wnt1*. In the future, it is planned to expand this network with other genes expressed at the MHB, including known modulators of the *Fgf8* signaling pathway such as the *Fgf* inhibitors *Sprouty* [35],[36], *Sef* [37] or *Mkp3* [38],[39]. In the present network these regulators at the signal transduction level are not considered. Being widely coexpressed with *Fgf8* , they are assumed to fine-tune *Fgf8* expression itself and the expression of *Fgf8* target genes by negative feedback modulations. This exquisite regulation of *Fgf8* agrees well with results demonstrating a concentration-dependent effect of *Fgf8* signaling [23],[40]. However, inclusion of these fine-tuning mechanisms would require a detailed kinetic model of *Fgf8* signaling at the MHB, for which we lack the mechanistic details. Also, such a model would not be compatible with the available coarse-grained data from *in situ* hybridization experiments. By restricting ourselves to regulations on the transcriptional level, we can treat the IsO network within phenomenological modeling environments, where a minimum of mechanistic detail is required.

The precise range of diffusion of the Fgf8 and Wnt1 molecules from the murine MHB is still an unresolved issue. With respect to Fgf8, we base our assumptions on the idea that secreted Fgf8, by binding to extracellular HSPGs or by active endocytosis, is a rather sticky protein that does not diffuse very far away from its source (mRNA expression) (see, for example, [29],[41]). This assumption is also backed up by the fact that the expression of direct gene targets of *Fgf8* signaling, such as the inhibitory proteins Sef, Spry and Mkp3, is centered around the *Fgf8* -positive domain and does not reach the di-/mesencephalic boundary rostrally or the r1/r2 boundary caudally [42]. The range of Wnt1 diffusion from the MHB is even less clear, as Wnts are (in contrast to Fgfs) lipid-modified secreted glycoproteins. These lipid-modifications make them hydrophobic and probably also membrane-tethered, so that they can only signal in the vicinity of the secreting cells [43]. The phenotype of the 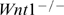 mutants, however, suggests that Wnt1 also acts as a long(er)-range signal, reaching at least the outer borders of the *Fgf8* -positive domain in the rostral hindbrain and of the *En1* -positive domain in the midbrain [10],[26]. In fact, recent data suggest that Wnts are transported as multimeric complexes through the extracellular space (reviewed by [43]).

In [18],[44] a functional equivalence and conserved biological function of *Pax2* and *Pax5* was shown. A similar functional equivalence and conserved biological function in MHB development was also shown for *En1* and *En2* and Drosophila *En* [45],[46]. Moreover, [18] and [45] have shown that the different phenotypes of the *En1* vs *En2* and *Pax2* vs *Pax5* mutants (with *En1* and *Pax2* mutants having a stronger phenotype than *En2* and *Pax5* mutants [18], [47]–[49]), are mostly due to the divergent spatio-temporal expression patterns of the *En* and *Pax* genes between the one- and eight-somite stages, and not due to their divergent biochemical properties. We have therefore decided to subsume *En1* and *En2* as well as *Pax2* and *Pax5* in one and to write *En* and *Pax* , respectively, in an initial attempt to simplify our models. Future expansions of these models will take into account the divergent spatio-temporal expression patterns and thus functional roles of these four genes in MHB development.

For our purposes herein, a one dimensional spatial model of the E10.5 neural tube suffices (see Methods). We therefore focus on the anterior–posterior dimension and do not take the dorso–ventral and medio–lateral directions into account. In particular, we neglect *Wnt1* expression in the dorsal and ventral midline of the midbrain/caudal diencephalon.

### Inference of regulatory interactions by minimization of Boolean functions

Previous work on the reverse engineering of biological networks has mainly focused on their reconstruction from time-courses of expression levels, see e.g. the reviews [50],[51] and references therein. Here, we demonstrate that also spatial expression patterns can be used for this purpose. Due to the scarcity of experimental data, reverse engineering approaches cannot reasonably aim at the complete identification of a regulatory network. Rather the challenge is to infer a maximum of regulatory interactions from the data. Under the Boolean network model, this is equivalent to the analysis of partially filled truth tables (see Methods). For this, two strategies are presented in this contribution. Their practicability is demonstrated by predicting several key genetic interactions at the MHB. However, the presented methodology is not restricted to this example and readily applicable to other pattern formation processes.

The first strategy is the identification of necessary interactions. In other words, we look for interactions included in all possible networks that are consistent with the data. This is a very strict condition. Consequently, only few predictions will result from this analysis; these, however, can be considered reliable. As in our example of the IsO network, the necessary interactions by themselves will typically not form a valid network explaining the data.

As a second strategy, we propose the determination of all minimal Boolean update functions. Here we assume that these minimal interactions constitute the core module of the unknown network. In the example of the IsO, this assumption is justified, as the minimal networks are fully backed up by experimental data. The condition that an interaction be included in a minimal network is weaker than that of being a necessary interaction. Consequently, our second strategy will typically yield additional predictions, that should be considered less reliable than the necessary interactions. In the IsO example, four necessary interactions could be found, while an additional six interactions were obtained from the minimality analysis. Hence, the analysis of minimal networks yielded valuable insights into the regulatory network between the IsO genes, which go beyond what could be obtained from determining all necessary interactions. A further advantage of the second strategy is that it leads to a functional network which is able to explain the data.

Standard ways of finding minimal Boolean functions are either Karnaugh-Veitch maps [52],[53] or the Quine-McCluskey algorithm [54],[55] — two functionally equivalent approaches. Karnaugh-Veitch maps are a clever graphical representation of Boolean functions. They transform the problem of finding minimal Boolean expressions into the problem of finding a maximal-size covering of either the *true* - or the *false* -entries. For small functions (typically no more than five inputs) the latter problem can be easily solved by inspection. For larger functions the Quine-McCluskey algorithm can be used instead, whose tabular form is less illustrative but easier to implement. However, also the Quine-McCluskey algorithm has a limited range of use, as it tries to solve a problem which has been shown to be NP-complete, cf. the Cook-Levine Theorem about the Boolean satisfiability problem [56]. Therefore, in the case of large systems, heuristic methods like the Espresso algorithm [57],[58] may be more suitable.

### The minimal vs the literature network

We observe that the literature network from Figure 2C is much more complex than the minimal networks shown in Figure 2B. This gives rise to the question why the more densely connected network apparently proved more favorable. To this end, it is interesting to study the different networks with respect to topological properties such as robustness [59]. A first result in this direction already becomes evident from the robustness analysis of the PDE model. There we found that for the densely connected network the expression domains of *En* and *Pax* become centered around the MHB even when started from perturbed initial conditions. Obviously, this is not the case for the minimal network since here *En* and *Pax* are seperated from the other genes. Their steady state expression domains consequently depend only on their initial conditions and are independent of the other genes. In particular, their expression domains will not be centered around the MHB. Hence, the additional regulations included in Figure 2C make the correct spatial alignment of the different expression domains robust against perturbations of the initial conditions.

Remarkable about the structure of the networks from Figure 2 is the high number of positive feedback loops, especially in the literature network from Figure 2C. It has been conjectured by [60] that this kind of feedback is required for a system to exhibit multistability. Over the last years this conjecture has been proven for large classes of biological systems [61]–[66]. Multistability is an essential property of regulatory networks involved in pattern formation, as they have to assume different steady states at different spatial positions. The role of positive feedback for spatial differentiation has been studied in [67].

Summing up our analyses of the literature network, we can describe the role of its single interactions as follows: The mutual inhibition of *Otx2* and *Gbx2* (interaction *(1)* in Figure 2C) is responsible for the division of the neural tube into an *Otx2*+/*Gbx2*− and an *Otx2*−/*Gbx2*+ territory. Opposing interactions *(2)* and *(3)* of *Otx2* and *Gbx2* restrict the expression of *Fgf8* and *Wnt1* to the *Otx2*−/*Gbx2*+ and *Otx2*+/*Gbx2*− territory, respectively. The mutual dependence between *Fgf8* and *Wnt1 (4)* confines their expression domains to the rostral and caudal side of the MHB, respectively. In particular, if simulating e.g. the PDE model without the predicted maintaining influence of *Fgf8* on *Wnt1* (not shown), the expression of *Wnt1* spreads up to the rostral end of the neural tube. Finally, a densely connected positive loop between *Fgf8*, *Wnt1*, *En* and *Pax (5)*–*(8)* ensures the stable maintenance of these genes' expression as well as their central alignment around the MHB.

### Spatial models of regulatory networks

In order to evaluate our IsO network we derived spatial dynamical models from it. Theoretical modeling has long been used to explain pattern formation in living organisms. The employed models range from coarse-grained multi-compartment Boolean [68],[69] and generalized logical models [70]–[72] over multi-compartment difference and (ordinary) differential equation models [73]–[75] to PDE models [76],[77].

In a first approach, we used a parameter-free Boolean model. Boolean models are generally applicable in a purely qualitative context and well able to deal with the exquisite, intricate mechanisms of gene regulation [78]. While they are often able to capture the essential characteristics of a system, their time-scale is arbitrary and their dynamics are very crude. This made it necessary to transform our Boolean model into a multi-compartment ODE and a PDE model (see Methods). These continuous models allow us to check for properties like the spatial refinement of expression domains and to introduce a rough time-scale. As transformations of the Boolean model, the ODE and PDE model are still phenomenological models. They allow for continuous simulations but do not reflect the underlying biochemical reactions better than the Boolean model. In particular, they model only the effective output of *Fgf8* and *Wnt1* signaling. The setup of a mass-action based model of the whole IsO network was infeasible due to our lack of information about mechanistic details.

In the PDE model, a critical point is the modeling of intercellular communication. In [28],[29] diffusion-based mechanisms are suggested for *Fgf8* and *Wnt1* signaling. These diffusion processes are typically modeled by expressions based on the so-called *Heat equation*. Together with terms for production and decay these expressions constitute reaction-diffusion PDEs. This type of equation is frequently used to model pattern formation in living organisms, see e.g. [79],[80] and, in particular, [81] for a reaction-diffusion model of *WNT* signaling. The Heat equation actually describes the distribution of heat in a given region over time. It is based on Fourier's law which states that the local flow rate of heat energy is proportional to the negative temperature gradient. We argue that, in our example, the Heat equation describes the diffusion of secreted proteins at least in a crude qualitative sense: Diffusion is directed from regions with high concentrations to regions with low concentrations and the rate of diffusion positively correlates with the difference in concentrations. Therefore, we consider the PDE model suitable to qualitatively test statements about the refinement and sharpening of expression domains.

The transformation of a Boolean model into a continuous model entails the introduction of kinetic parameters. Especially in the context of pattern formation it has been shown that a network's function is carried out robustly against perturbations of these kinetic constants [75],[82]. While this result does not allow us to completely ignore quantitation in these models, it demonstrates the importance of a network's structure for its functionality. Along those lines, we investigated also the robustness of our models. We found that none of the observed behaviors of the ODE and PDE models is specific to a certain parameter set. As discussed above, some mild restrictions need to be imposed on the parameter space in order to ensure a robust functionality of the models. Other than that, parameters can be chosen from a large range.

Our modeling pipeline — from Boolean over ODE to PDE models — exemplifies how more and more detailed models of regulatory networks can be built from qualitative information. The transformation methods that we used (see Methods) ensure that all models reflect the experimentally validated interactions from Figure 2C.

### Possible model extensions

Apart from the previously mentioned extensions of our models (to include *Fgf* signaling inhibitors and a more refined picture of *Engrailed* and *Pax* expression patterns and interactions), other important genes for MHB establishment, IsO function and mid-/hindbrain development such as *Lmx1b* [83]–[85] or *Grg4* [86],[87] will be included in future versions. Possible extensions of our models will also aim at including related processes, such as the specification of cell populations in the patterning field. The MHB not only controls patterning of the mid- and rostral hindbrain, but also controls the location and size of the midbrain dopaminergic and rostral hindbrain serotonergic neuronal populations [88]. Finally, one could also take into account the dorso-ventral patterning of the neural plate/tube, which would require a spatially higher dimensional model, based e.g. on multi dimensional information from our *IDGenes* database.

### Conclusions

We demonstrated that similar to temporal expression patterns, also spatial expression patterns can be used to gain information about the structure of regulatory networks. In particular, we showed that the characteristic expression patterns of key IsO genes reveal a maintaining effect of Fgf8 on *Wnt1* expression — a prediction we also validated experimentally. The presented method recommends itself to be applied to other patterning formation processes. It allows to harness qualitative experimental results like snapshots of expression patterns for model construction in Systems Biology. This is exemplified by our spatial models of the MHB. These are competent to reproduce time-courses of expression patterns both under wild-type and various knock-out conditions, which makes them promising starting points for further investigations.

## Methods

### Ethics statement

Animal treatment was conducted under federal guidelines for the use and care of laboratory animals and was approved by the HMGU Institutional Animal Care and Use Committee.

### Database implementation

*IDGenes* is implemented as a relational database using PostgreSQL (http://www.postgresql.org/) (see Text S1). Gene expression and gene interaction data were carefully derived from literature and stored in the database. *IDGenes* is accessible via standard HTML. Moreover, an optional Java web interface was developed in order to visualize gene expression in the embryonic mouse brain. 3D graphics of the anatomical brain areas for different developmental stages were created with the open source software Blender (http://www.blender.org/) and integrated into *IDGenes*. Via the web interface an anatomical area and developmental stage can be set and the corresponding information about gene expression as well as genetic interactions can be retrieved from the *IDGenes* database (see Text S1). Further instructions for the use of the database are available on the help-page, which can be accessed via the help-button in the left frame of the *IDGenes* website.

### Setup of modeling environment

We think of the neural plate/tube as a chain of cells, which are able to communicate via *Fgf8* and *Wnt1* signaling. Since all cells in one of the six compartments I–VI from Figure 1 face the same conditions with respect to *Fgf8* and *Wnt1* signaling and show the same expression pattern we can subsume them into one compartment. Of course, these compartments do not have to be equally long, i.e. they can contain different numbers of cells. So, our neural plate/tube consists of six compartments 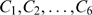, in each of which the same regulatory network between the genes *Otx2*, *Gbx2*, *Fgf8*, *Wnt1*, *En* and *Pax* is working. To specify this network we introduce a set of *template variables* 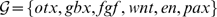 and assign to each an update rule. To account for inter-cellular communication we also introduce template variables 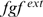 and 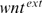 indicating if, respectively, secreted Fgf8 or Wnt1 protein is present, and let our update rules depend on these variables instead of *fgf* and *wnt* . For 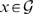 we let 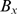 denote the respective update rule and 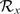 the set of inputs of 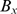, i.e.
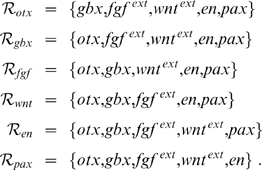
The introduction of template variables allows us to specify the network as a simple one-compartment regulatory network. From this one-compartment network we can now easily build up a multi-compartment model of the MHB. In each compartment 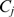, 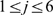, we have copies of our template variables denoted by a subscript 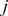. Hence, 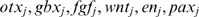 indicate if the corresponding gene is expressed in compartment 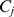 or not, and 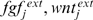 indicate if secreted Fgf8 or Wnt1 protein is present in compartment 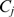. To complete the definition of our network, we just need to specify the relation between the variables 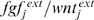 and the variables 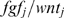. In the following, we simply assume that secreted Fgf8/Wnt1 protein is present in a compartment if *Fgf8*/*Wnt1* is expressed in the compartment itself or in any adjacent compartment. Hence for these species we have the relations
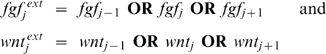
(2)for 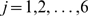, where formally 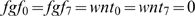. This reflects the fact that the transcription factors *Otx2*, *Gbx2*, *En* and *Pax* have cell-autonomous roles, whereas *Fgf8* and *Wnt1* function non-cell-autonomously via diffusion of secreted protein.

Now given update functions 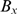, 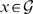, our model is determined by the synchronous update rule
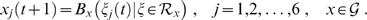
(3)

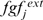
 and 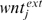 are then also determined by (2).

### Logical analysis of gene expression patterns

Our goal is now to find update rules, such that model (3) has a steady state pattern
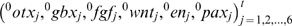
identical to the left-hand table in Figure 1C. We denote this condition by (C). The resulting steady state values of the variables 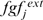 and 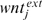 are denoted by 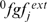 and 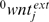, respectively. Clearly in this steady state
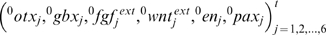
is identical to the right-hand table in Figure 1C.

Now, each of our update functions 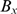, 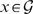, depends on five variables and can thus be represented as a truth table with 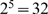 entries. The introduction of the template variables 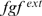 and 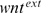 allows to consider the compartments separately. Hence, in terms of the Boolean update functions, condition (C) is equivalent to
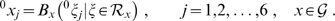
(4)As long as these conditions are not contradictory, this allows us to fix at most six entries in the truth table of each update rule. The remaining entries are indetermined, so-called don't cares. This shows that multiple Boolean update rules satisfy condition (C). Therefore, we restrict our analysis to the detection of key interactions.

In a first step, we determine all non-trivial dependencies in the truth tables. Species 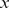 depends non-trivially on its input 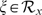 if there is an assignment of truth values to the inputs of 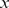 such that a change of 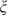 keeping all other inputs constant changes the output of 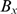. In this case, we say that there is a necessary interaction between 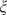 and 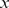, since, if this interaction was missing, the change in the output of 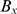 could not be explained. We call the interaction positive, if a change of 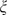 from *true* to *false* changes 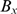 also from *true* to *false* , otherwise the interaction is called negative. We conduct an exhaustive search of the partially filled truth tables for necessary interactions. More precisely, we look for all pairs of (determined) entries with different outputs that differ in exactly one input. This way, we find that 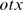 and 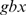 depend non-trivially and negatively on each other. Moreover, 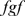 depends non-trivially and positively on 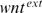 and, vice versa, 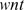 on 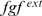.

In a next step, we look for minimal Boolean expressions describing the partially filled truth tables. In our example, we find the minimal Boolean expressions given in (1) by using the Karnaugh-Veitch maps shown in Text S1. Note that in (1) we write non-italic protein names instead of the superscript ext for better readibility. In the case of *Fgf8* and *Wnt1* two equally minimal expressions can be found, the two possibilities are given in brackets.

Note that in our modeling environment any auto-regulation is excluded. We can now see why this restriction is necessary. Otherwise, the method outlined above would always yield the trivial minimal solution, where each species 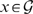 positively regulates itself, i.e. 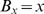.

### Explant cultures and bead implantations

Explant cultures of anterior neural plates/tubes (open-book preparations) from wild-type (C57BL/6) mouse embryos were essentially prepared as reported by [89]. Bead preparation is described in [90].

### Whole mount *in situ* hybridization (WISH)

Explants were fixed in fresh 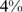 paraformaldehyde (PFA) for 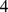 hours at 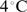 and WISH was carried out using standard procedures.

### Modeling the regulatory network

From the network shown in Figure 2C we derived Boolean update rules, where the synergy of Fgf8 and Wnt1 in the activation of *En* was modeled using OR gates. Stated in terms of the template variables these Boolean functions read
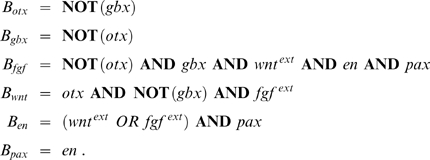
By implanting these concrete update rules into the modeling environment specified by (3) we obtain a multi-compartment Boolean model of the IsO.

This model was subsequently transformed into an ODE model consisting of the now continuous variables 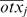, 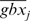, 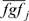, 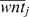, 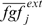, 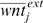, 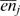, 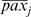 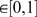, 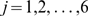. The temporal development of the variables 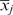, 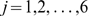, 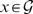, is governed by the ODEs
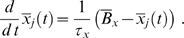
The right-hand side of these equations consists of two parts, an activation function 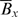 describing the production of the species 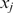 and a term for decay which we assume to be proportional to 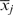. Note that due to the normalization of concentrations to the unit interval, we have equal production and decay rates described by the parameters 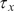. For the mathematical details of the transformation process we refer the interested reader to [91]. The crucial point is how we define the continuous activation functions 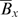. In [92] a method is introduced that allows to obtain the 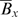 directly from the Boolean update rules 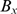. The basic idea is to replace Boolean step functions by sigmoid (positive) Hill functions 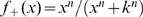 with parameters 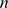 and 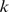. The Boolean operators **NOT**, **AND** and **OR** are then replaced by continuous expressions as shown in Figure 6: **NOT** is substituted for by a negative Hill function 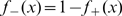; the continuous homologues of the **AND** and **OR** gates are obtained by an interpolation technique. Using this transformation, we obtain, for instance,
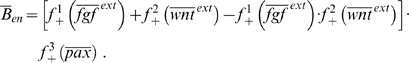
Here 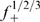 denote positive Hill functions with different parameters 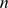 and 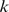. With regard to the species 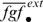 and 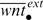, the relations (2) are replaced by
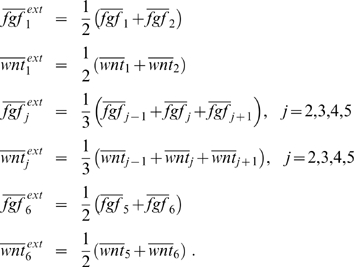



In order to analyze spatial effects in a continuous manner, the multi-compartment model was transformed into a reaction-diffusion PDE model. The neural plate/tube is now no longer coarse-grained into compartments and we no longer add a subscript 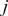 to the variables in order to indicate their spatial position. Instead we let our variables depend not only on time but also on a continuous spatial coordinate 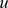. The PDE model is given by reaction-diffusion equations of the form
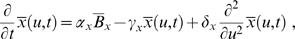


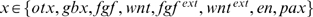
 with production rates 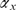, decay rates 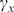 and diffusion constants 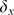. Except for the species 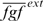 and 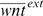 we use the activation functions 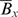 from above and set 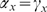 as well as 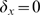. Hence these equations simplify to the ones from the multi-compartment ODE model. In the equations for 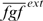 and 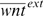 we have 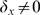 and assume that at a position 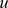 production of secreted protein is proportional to the amount of mRNA present at 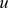. Hence we have
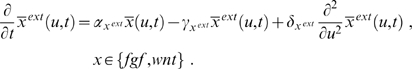
Ready-to-use MATLAB (http://www.mathworks.com) implementations of the models can be found in Dataset S1 and Dataset S2.

## Supporting Information

Text S1 Supplementary text
(3.01 MB PDF)

Video S1 Simulation of the PDE model under wild-type conditions
(0.60 MB MP4)

Video S2 Simulation of the PDE model under *Fgf8* ^−/−^ mutant conditions
(0.34 MB MP4)

Video S3 Simulation of the PDE model under *Wnt1* ^−/−^ mutant conditions
(0.33 MB MP4)

Video S4 Simulation of the PDE model under *En* ^−/−^ mutant conditions
(0.26 MB MP4)

Video S5 Simulation of the PDE model under *Pax* ^−/−^ mutant conditions
(0.24 MB MPG)

Dataset S1 ODE model as MATLAB .m file
(0.01 MB TXT)

Dataset S2 Archive containing MATLAB files for simulation of the PDE model
(4 KB ZIP)
