# Supplementary material for: Spatial Analysis of Expression Patterns Predicts Genetic Interactions at the Mid-Hindbrain Boundary
Source: PLoS Comput Biol. 2009 Nov 20;5(11):e1000569. doi: 10.1371/journal.pcbi.1000569 (PMC2774268; doi:10.1371/journal.pcbi.1000569)
Supplement: Text S1 — Supplementary text (3.01 MB PDF) [file pcbi.1000569.s001.pdf]

Spatial analysis of expression patterns predicts genetic interactions at the mid-hindbrain boundary — Supplementary information

Dominik M. Wittmann[*†], Florian Blöchl[*], Dietrich Trümbach[‡§], Wolfgang Wurst[‡§], Nilima Prakash[‡] and Fabian J. Theis[*†¶]

October 16, 2009

Besides this supplementary text the online supplement contains the following files:

Video S1: simulation of the PDE model under wild-type conditions

Video S2: simulation of the PDE model under *Fgf8*$^{-/-}$ mutant conditions

Video S3: simulation of the PDE model under *Wnt1*$^{-/-}$ mutant conditions

Video S4: simulation of the PDE model under *En*$^{-/-}$ mutant conditions

Video S5: simulation of the PDE model under *Pax*$^{-/-}$ mutant conditions

Dataset S1: ODE model as MATLAB `.m` file

Dataset S2: archive containing MATLAB `.m` files for simulation of the PDE model

[*]Computational Modeling in Biology, Institute for Bioinformatics and Systems Biology, Helmholtz Centre Munich, German Research Centre for Environmental Health, 85764 Munich-Neuherberg, Germany

[†]Zentrum Mathematik, Technische Universität München, 85747 Garching, Germany

[‡]Molecular Neurogenetics, Institute of Developmental Genetics, Helmholtz Zentrum München, German Research Centre for Environmental Health, Technische Universität München, Deutsches Zentrum für Neurodegenerative Erkrankungen (DZNE), 85764 Munich-Neuherberg, Germany

[§]Max Planck Institute of Psychiatry, 80804 Munich, Germany

[¶]Max-Planck-Institute for Dynamics and Self-Organization, 37077 Göttingen, Germany

Contents

1 **Expression patterns of the IsO genes at E10.5 (ad main Figure 1)** 2

2 ***IDGenes* database** 2

3 **Minimization of Boolean functions using Karnaugh-Veitch maps** 2

4 **Parameter choice for model simulations (ad main Figures 4 and 5)** 4

5 **Sampling of initial conditions and classification of steady states (ad main Figure 4)** 4

6 **Simulations of the PDE model (ad main Figure 5)** 6

7 **Simulations of further LOF experiments** 7

8 **Parameter analysis of the ODE model (ad main Figure 7)** 7

9 **Parameter and robustness analysis of the PDE model** 10

1 Expression patterns of the IsO genes at E10.5 (ad main Figure 1)

The expression pattern shown in main Figures 1A and 1B was derived from various *in situ* hybridization experiments, for a review see e.g. [5] and, in particular, Figure 1c therein. Further details can be found in the primary research publications listed in Table 1 and in our *IDGenes* database.

Table 1: Primary research publications investigating the expression pattern shown in main Figure 1.

Gene	PubmedID	Author, year	PubmedID	Author, year
Otx2	11231064	Garda et al., 2001	1353865	Simeone et al., 1992
	8101484	Simeone et al., 1993		
Gbx2	11231064	Garda et al., 2001	9247335	Wassarman et al., 1997
Fgf8	7768185	Crossley and Martin, 1995	9056772	Lee et al., 1997
Wnt1	7577673	Rowitch and McMahon, 1995	2907320	Davis and Joyner, 1988
	3594565	Wilkinson et al., 1987	8275860	Parr et al., 1993
En1	2907320	Davis and Joyner, 1988	7577673	Rowitch and McMahon, 1995
En2	2454212	Davis et al., 1988	2907320	Davis and Joyner, 1988
Pax2	10934015	Bouchard et al., 2000	1977575	Nornes et al., 1990
	7577673	Rowitch and McMahon, 1995		
Pax5	7577673	Rowitch and McMahon, 1995	1283313	Asano and Gruss, 1992

2 *IDGenes* database

Figure S1 shows a screenshot of the web interface and the relational database scheme of *IDGenes* (`http://www.helmholtz-muenchen.de/idgenes`).

3 Minimization of Boolean functions using Karnaugh-Veitch maps

Here we outline how minimal Boolean expressions for partially filled truth tables can be found by using Karnaugh-Veitch (KV) maps. No rigorous mathematical explanations are given but the interested

A

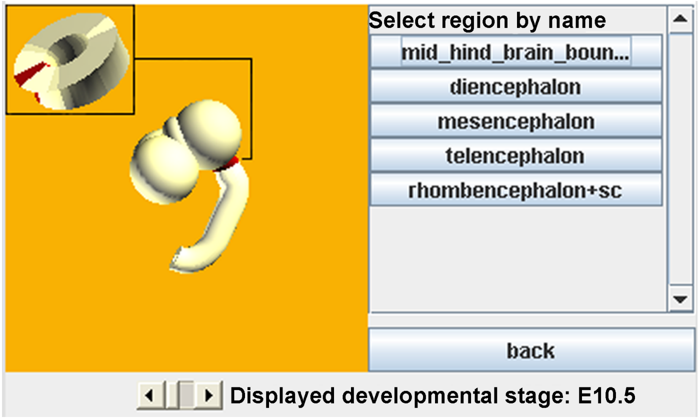

B

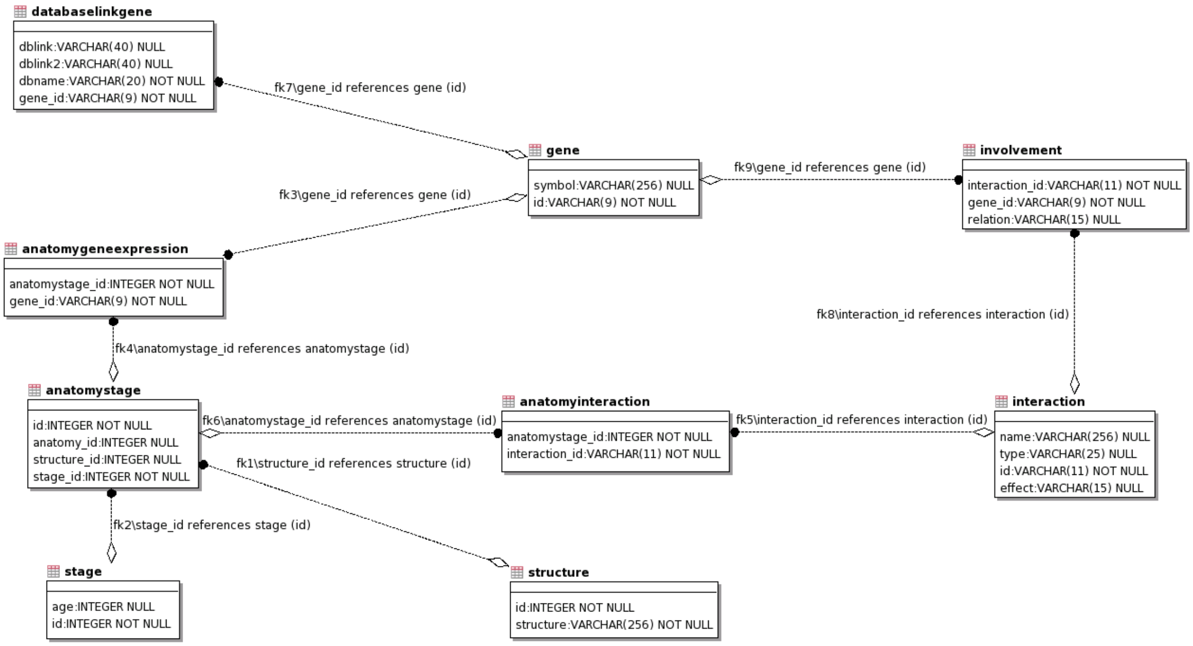

Figure S 1: **A** Screenshot of a Java applet embedded in the web interface of the *IDGenes* database. An anatomical area can be selected by the buttons on the right side. In the picture, 'mid_hind_brain_boundary' is chosen which corresponds to the red area in the mouse brain on the left. The anatomical brain components are hierarchically ordered, i.e. after selection of a specific area an enlarged 3D model of the structure is shown. An example is displayed for the MHB (and the selected floor plate area, red) within the box in the upper left-hand corner. **B** Relational database scheme of *IDGenes*. The entity relationship diagram was generated using the software *Together* from Borland.

reader is referred to [1, 3].

Each of our Boolean update functions B_x, $x \in \mathcal{G}$, can be represented as a truth table with $2^5 = 32$ entries. Condition (4) from the main text allows to specify at most six entries in each truth table. The remaining entries are indetermined 'don't cares'. These truth tables are now represented as KV maps, see Figure S2. Actually, these maps are three dimensional cubes but for better presentability the cube was sliced up and the two layers put next to each other. Moreover, the green as well as the blue sides are identified, so each 'slice' is actually a torus. By inspection, we now determine a covering of the *true*-entries by rectangular boxes of size $2, 4, 8, 16$ or 32 such that

- No *false*-entry is contained in any of the boxes.
- All *true*-entries are contained in at least one box.
- The boxes have maximum size.
- The number of boxes is minimal.

Note that there may be multiple coverings satisfying these conditions. In Figure S2 all suitable coverings are shown. From each box in these coverings a conjunction term (AND gate) is built, where variables appearing both, negated and non-negated within the box, are omitted. These conjunction terms are linked by disjunction (OR gate) and give a minimal so-called disjunctive normal form (DNF) of the Boolean expression. If one wants to find the dual minimal conjunctive normal form (CNF), i.e. a series of disjunction terms linked by conjunction, one applies the above procedure to the inverted Boolean function. From the minimal DNF of the inverted Boolean function a minimal CNF of the original Boolean function can then be obtained by inversion and application of the *De Morgan's laws.*

We illustrate this using B_{fgf} as a showcase. Two coverings satisfying the above mentioned conditions could be found, each consisting of only one box, cf. the dashed and solid red boxes in Figure S2B. Let us choose the first (dashed lines). Here all variables except *otx* and *wnt* appear both, negated as well as non-negated. Consequently, the conjunction term for this box is **NOT** (*otx*) **AND** *wnt*. Similarly, the other expressions from equation (1) in the main text can be deduced from the maps in Figure S2.

4 Parameter choice for model simulations (ad main Figures 4 and 5)

When simulating our continuous models we do not intend to fit quantitative time-courses of concentration levels. Rather we want to check if our models exhibit certain (more qualitative) behaviors, like the stable maintenance of a specific expression pattern. In Figures 4 and 5 of the main text the following ad-hoc parameters were used:

ODE model Hill exponents $n = 5$; thresholds (affinities) $k = 0.1$; life-times $\tau_x = 1$.

PDE model For $x \in \{fgf^{\text{ext}}, wnt^{\text{ext}}\}$: production rates $\alpha_x = 1$; decay rates $\gamma_x = 0.8$; diffusion rates $\delta_x = 0.01$. For all other species: decay and production rates $\alpha_x = \gamma_x = 1$; diffusion rates $\delta_x = 0$. Note that, in the case of positive diffusion rates δ_x, we assume a lower decay rate of protein than of mRNA. This way, the model's qualitative behavior does not change within a $\pm 10\%$-strip around the ad-hoc parameter values.

For the simulation of the LOF experiments the production rate of the knocked-out gene was set to zero.

5 Sampling of initial conditions and classification of steady states (ad main Figure 4)

This section refers to Figure 4 of the main text. For details on main Figure 7, see supplement section 8.

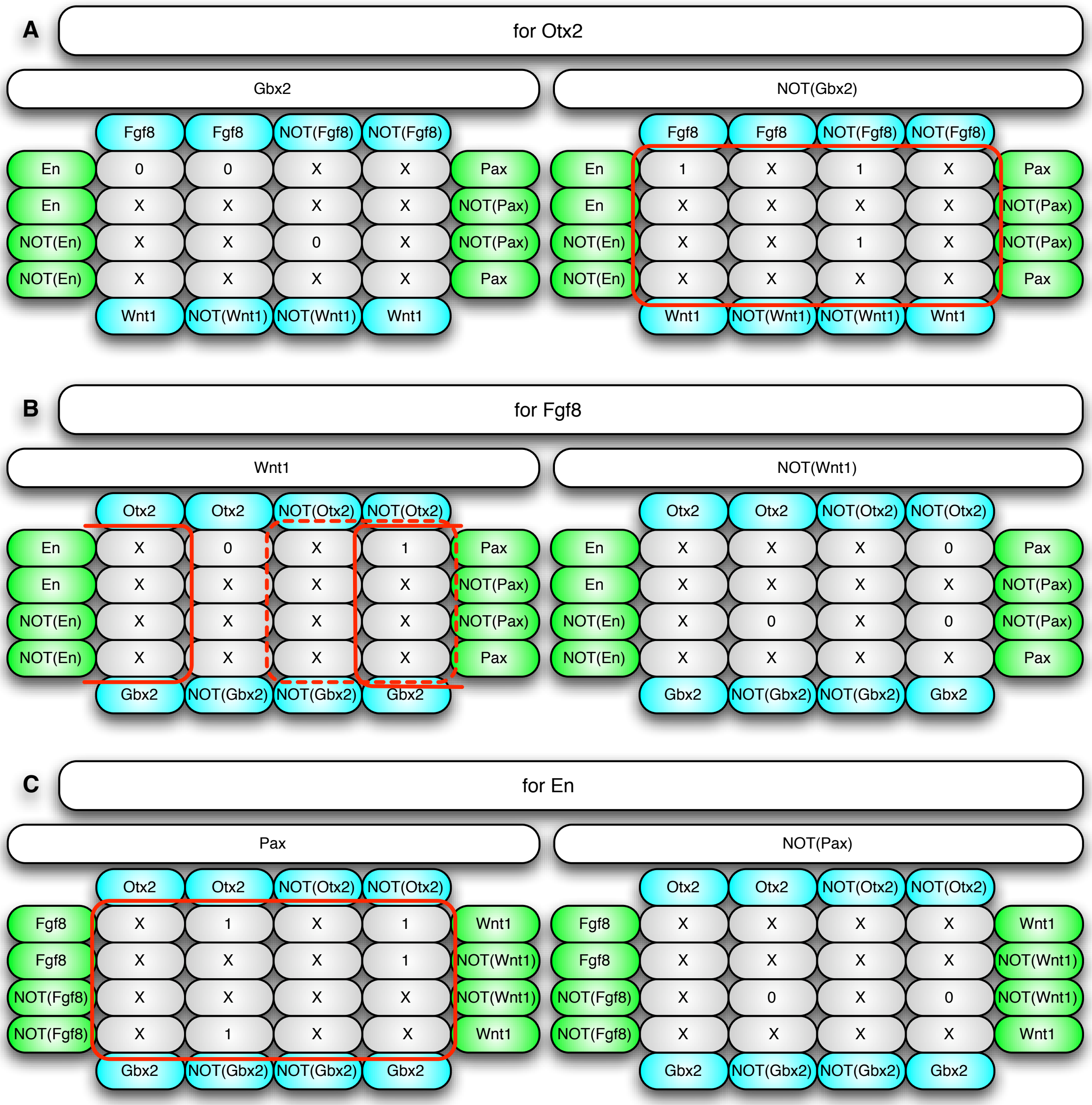

Figure S2: KV maps for **A** *Otx2*, **B** *Gbx2* and **C** *En*. 0: *false*, 1: *true*, X: 'don't cares'. In each map the blue and green sides are identified. Red boxes indicate the maximal coverings. Similar maps for *Gbx2*, *Wnt1* and *Pax* can be obtained by flipping *Gbx2* and *Otx2* in A, *Fgf8* and *Wnt1* in B and *En* and *Pax* in C, respectively.

Table 2: Boundary conditions for the PDE model.

	left ($u = 0$)	right ($u = 1$)
otx	1	0
gbx	0	1
fgf	0	0
fgf^{ext}	0	0
wnt	0	0
wnt^{ext}	0	0
en	0	0
pax	0	0

The multi-compartment ODE model was solved numerically with MATLAB `ode15s` (`http://www.mathworks.com`), a variable order multi-step solver. The required `.m` file is provided in Dataset S1. In the computational experiment shown in main Figure 4, the initial conditions in each run were determined by eight values: the levels of *Otx2* and *Gbx2* on the anterior as well as posterior side of the boundary and the levels of *Fgf8*, *Wnt1*, *En* and *Pax*, whose initial expression domains were all set to the four central compartments. The initial conditions of otx and gbx were sampled uniformly from $[0, 1]$. The initial conditions of the other variables were sampled such that the minimum of their 10-logarithms is uniformly distributed on $[-4, 0]$. To this end, we first sampled this minimum, let us call it a, uniformly from $[-4, 0]$. Then we randomly chose a variable and assigned it the initial condition 10^a. Finally, the 10-logarithms of the three remaining variables were sampled uniformly from $(a, 0]$. Each simulation was run until the maximum distance beween a variable's values at two successive time-points dropped below 10^{-6}. In the last expression pattern a variable was considered to be expressed at a certain position if its value was above its Hill threshold k. In the 10^5 runs only the six (discretized) steady states shown in main Figure 4A were reached.

6 Simulations of the PDE model (ad main Figure 5)

The PDE model was solved numerically with MATLAB `pdepe`, a solver for initial-boundary value problems for parabolic-elliptic PDEs in 1-D. The spatial coordinate u was normalized to the unit interval. Table 2 gives the boundary conditions.

In the wild-type simulation, initial conditions at $t = 0$ were chosen to mimic the fuzzy expression patterns at E8.5 (cf. upper Figure 5A of the main text). They were obtained by specifying the boundaries as well as the maximum of the expression profile of each species and interpolating these points by cubic splines. In detail, the points were (first coordinate = spatial position u, second coordinate = initial concentration):

***otx*:** $(0, 1), (0.5, 0), (1, 0)$

***gbx*:** $(0, 0), (0.5, 0), (1, 1)$

***fgf*:** $(0, 0), (0.45, 0), (0.625, 1), (0.8, 0), (1, 0)$

fgf^{ext}: $(0, 0), (0.45, 0), (0.625, 1), (0.8, 0), (1, 0)$

***wnt*:** $(0, 0), (0.2, 0), (0.375, 1), (0.55, 0), (1, 0)$

wnt^{ext}: $(0, 0), (0.2, 0), (0.375, 1), (0.55, 0), (1, 0)$

***en*:** $(0, 0), (0.25, 0), (0.425, 1), (0.6, 0), (1, 0)$

***pax*:** $(0, 0), (0.3, 0), (0.475, 1), (0.65, 0), (1, 0)$

For the simulation of the different LOF experiments, the initial condition of the wild-type simulation was used and the expression of the knocked-out gene was deleted. Also the production rate of this gene was set to zero.

In the limit $t \to \infty$ a discontinuity at the MHB ($u = 0.5$) arises in our simulations. Therefore, a fine grid of evaluation points around $u = 0.5$ is necessary to guarantee numeric stability. All files needed for the integration using MATLAB `pdepe` are contained in the archive Dataset S2.

7 Simulations of further LOF experiments

Figure S3 shows simulations of $Fgf8^{-/-}$, $En^{-/-}$ and $Pax^{-/-}$ mutants.

8 Parameter analysis of the ODE model (ad main Figure 7)

Here we give technical details and provide additional information on the robustness analysis shown in Figure 7 of the main text. First, we analyze the *Otx2*–*Gbx2* switch. Consider a mutual inhibition modeled as

$$\begin{aligned} \dot{x}_1 &= \frac{k_1^n}{x_2^n + k_1^n} - x_1 \\ \dot{x}_2 &= \frac{k_2^n}{x_1^n + k_2^n} - x_2 \, . \end{aligned} \tag{1}$$

For $n > 1$ three steady states are possible: The two stable steady states x_1 high/x_2 low (color coded red in the following), x_2 high/x_1 low (color coded blue) and an unstable steady state where x_1 and x_2 are expressed at medium levels (color coded magenta). The latter is irrelevant for biological systems, since minimal fluctuations will drive the system away from this state and into either of the two stable steady states. We now study how the sizes of the basins of attraction vary under parameter changes. To this end, we fix $k_2 = 0.1$ and vary $k_1 = i/1000$, $i = 1, 2, \ldots, 1000$. For each parameter configuration we evaluate the model starting at the points of the regular grid with cell size 0.1 until convergence. We count the points leading to the red, blue and magenta steady states and use these numbers as measures for the size of the basins of attraction. The results are displayed in Figure S4. Note that we have a total of 121 grid points. For $k_1 = 0.1$, i.e. for symmetrical parameters, the red and blue basins of attraction are equally large. Here, for identical initial conditions the system converges towards the unstable magenta steady state. In all other simulations the unstable steady state is not reached as no grid point is located in its one dimensional basin of attraction. For $k_1 \neq 0.1$ the switch becomes unbalanced and for $k_1 \ll 0.1$ or $k_1 \gg 0.1$ it exhibits an almost monostable behavior. This analysis indicates that for a functional switch the two parameters k_1 and k_2 need to be at least of the same magnitude.

After this preliminary analysis, we investigated the influence of the parameters on the ODE model of the IsO network. $3 \cdot 10^5$ simulations of the ODE model were run until convergence (threshold 10^{-9}). For better statistics, the number of runs was increased from 10^5 (as in main Figure 4) to $3 \cdot 10^5$ in order to account for the additional degrees of freedom in the sampling process. For the two threshold parameters describing the mutual inhibition of *Otx2* and *Gbx2* three different sampling schemes were used. For the remaining parameters, each n was sampled uniformly from $[2, 10]$, each $\log_{10}(k)$ uniformly from $[-3, 0]$ and each τ uniformly from $[0.5, 10]$. Similar ranges were used in previous studies [4, 2]. In main Figure 7A the two switch thresholds are fixed at their ad-hoc values from section 4. In main Figure 7B, their 10-logarithms are sampled uniformly from $[-0.9, -1.1]$, i.e. from a $\pm 10\%$-strip around their ad-hoc values. In main Figure 7C they are sampled as randomly as all other threshold parameters.

The initial conditions were sampled essentially as described in section 5. They are again determined by eight values: the levels of *Otx2* and *Gbx2* on the anterior as well as posterior side of the boundary and the levels of *Fgf8*, *Wnt1*, *En* and *Pax*, whose initial expression domains were all set to the four central compartments. The initial concentrations of *Otx2* and *Gbx2* were again sampled from $[0, 1]$. The initial

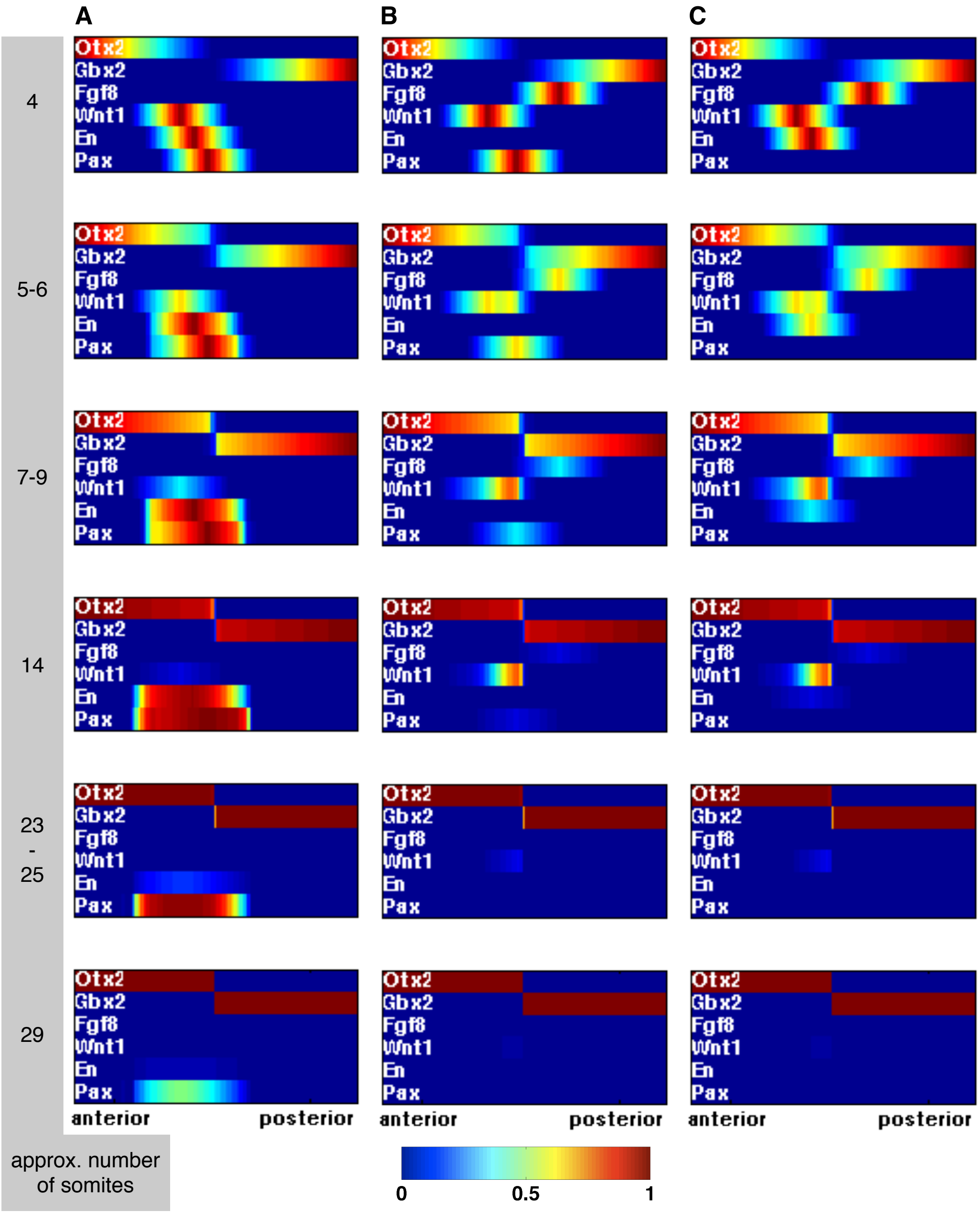

Figure S3: Simulations of a **A** *Fgf8*$^{-/-}$, **B** *En*$^{-/-}$ and **C** *Pax*$^{-/-}$ mutant using the PDE model. In lack of quantitative data ad-hoc parameters were used, see supplement section 4. As in the main text a relation of 1 somite per every 5 time units is assumed and gene expression domains are shown at time points $t = 1, 9, 21, 51, 101, 126$.

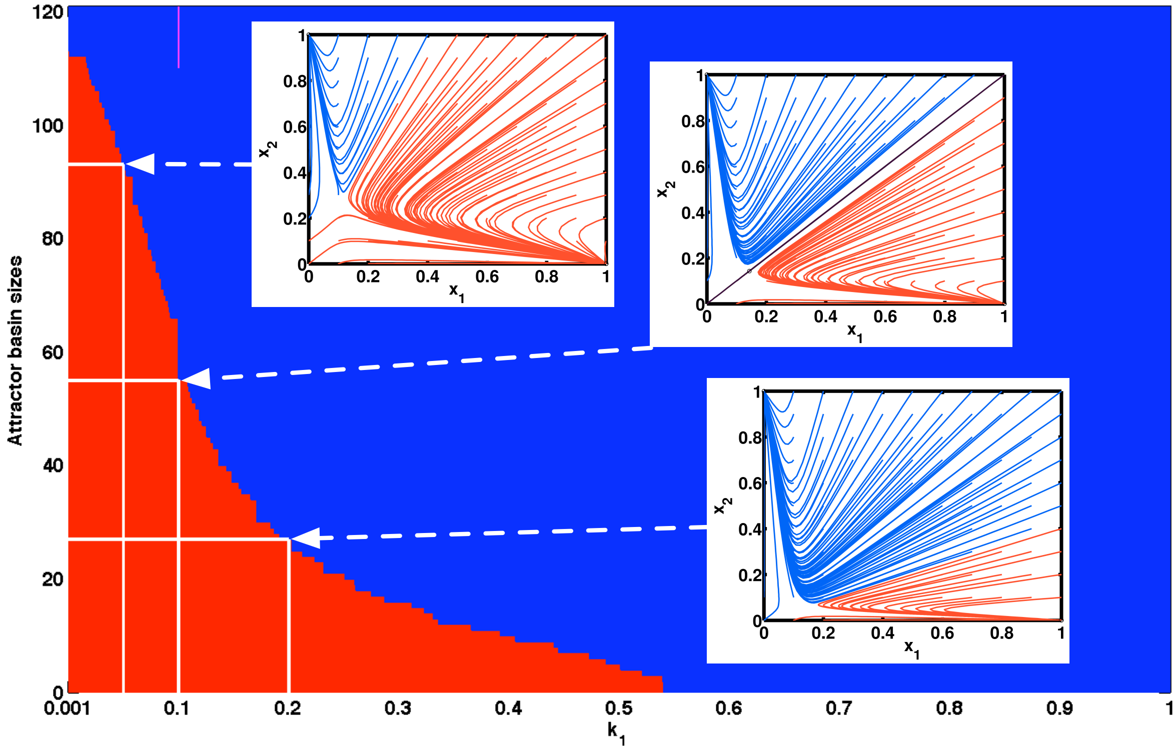

Figure S4: Basins of attraction of the mutual inhibition switch (1). Parameters $n = 5$ and $k_2 = 0.1$ are fixed, and $k_2 = i/1000$, $i = 1, 2, \ldots, 1000$ varies. For each configuration model (1) was evaluated until convergence (threshold 10^{-6}) starting at the points of the regular grid with cell size 0.1 as shown in the three insets. The number of points leading to the x_1 high/x_2 low (x_2 high/x_1 low) steady state is shown in red (blue). For $k_1 = 0.1$, the 11 points on the $x_1(0) = x_2(0)$ diagonal lead to an unstable steady state shown in magenta. The three insets show the model's phase planes for $k_1 = 0.05, 0.1, 0.2$.

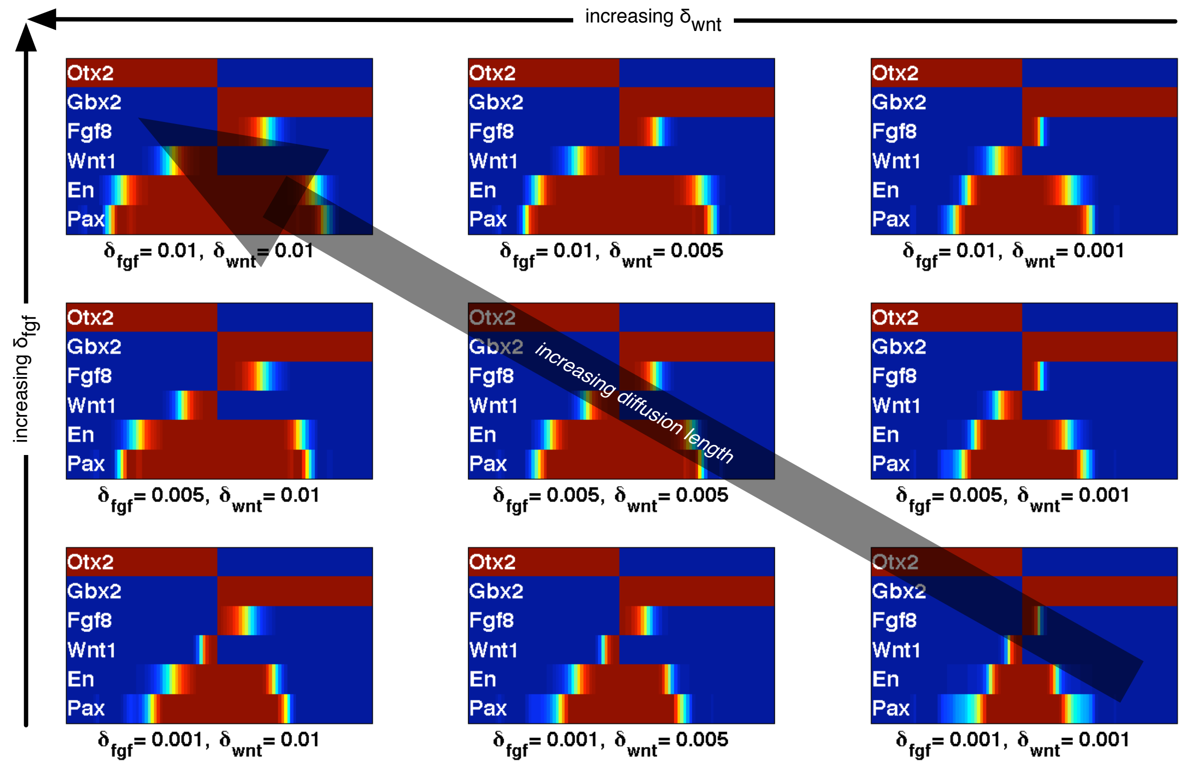

Figure S5: Simulation of the PDE model for different diffusion constants δ_{fgf} and δ_{wnt}. Other parameters are set to their ad-hoc values from section 4. Shown are the expression patterns of the IsO genes at time-point $t = 156$, which corresponds to approximately 35 somites (E10.5). With increasing δ_{fgf} and δ_{wnt} the length of the diffusion domains of Fgf8 and Wnt1, respectively, increases. Due to the mutual positive regulation of *Fgf8* and *Wnt1* this entails a broadening of the expression domains of *Wnt1* and *Fgf8*, respectively.

conditions of *Fgf8*, *Wnt1*, *En* and *Pax* were sampled such that the minimum of their 10-logarithms is uniform on $[-6, 0]$. For this the same sampling scheme as in section 5 was used. Note that the range of these initial conditions was adapted from $[10^{-4}, 1]$ (as in main Figure 4) to $[10^{-6}, 1]$ as we no longer have all $k = 0.1$ but $10^{-3} \leq k \leq 1$.

The discretization of the steady states is critical. Given a parameter configuration, we determined for each species the minimal as well as the maximal threshold k of its out-going interactions. We define that a species is 'expressed' if its value is above the maximal k and 'not expressed' if its value is below the minimal k. Hence, a species is expressed if it is fully active within the network and not expressed if it is completely inactive. This classification scheme cannot be applied to values between the minimal and maximal k. As any classification of such values into binary on-off categories is arbitrary and given that such values occurred in only 5.0% of all simulations we decided to simply ignore these runs. In the remaining runs, again only the six discretized steady states shown in main Figure 4A were reached.

9 Parameter and robustness analysis of the PDE model

Figure S5 shows the effect of varying diffusion constants δ_{fgf} and δ_{wnt}.

In order to investigate the effect of perturbed initial conditions, 100 initial conditions were sampled as follows: As in section 6, we define the initial condition as a spline interpolation of points. For each

gene the boundary conditions (the points with spatial coordinates $u = 0$ and $u = 1$) are chosen as in section 6. The coordinates of the interior points specifying the expression domains are uniformly sampled from a $\pm 10\%$-strip around the values used in section 6. Thus we obtained 100 perturbations of the initial conditions from section 6. For each of them the PDE model was simulated until convergence using the ad-hoc parameters. For each resulting steady state we first detected the position b of the *Otx2*–*Gbx2* interface. Over the 100 runs b varied between 0.44 and 0.61. Hence the initial conditions have a crucial effect on the position of the MHB along the anterior-posterior axis of the neural plate/tube. Subsequently, the steady states were compared to the steady state obtained for the unperturbed initial conditions from section 6. Both steady states were aligned at the *Otx2*–*Gbx2* interface and the integral over the absolute difference was numerically computed for each variable. In 99 out of the 100 runs none of these integrals was larger than 0.10. Only in one run a change of the qualitative behavior of the model could be observed. Here the expression of *Fgf8*, *Wnt1*, *En* and *Pax* was lost over time.

References

[1] Karnaugh M (1953) The map method for synthesis of combinational logic circuits *Transactions of the American Institute of Electrical Engineers* **72**(9): 593–599

[2] Ma W, Lai L, Ouyang Q and Tang C (2006) Robustness and modular design of the Drosophila segment polarity network *Mol Syst Biol* **2**(70)

[3] Veitch E (1952) A chart method for simplifying truth functions in *Proceedings of the 1952 ACM national meeting (Pittsburgh)* ACM New York, NY, USA 127–133

[4] von Dassow G, Meir E, Munro E and Garett M (2000) The segment polarity network is a robust developmental module *Nature* **406**(6792): 188–192

[5] Wurst W and Bally-Cuif L (2001) Neural plate patterning: Upstream and downstream of the isthmic organizer *Nat Rev Neurosci* **2**(2): 99–108
